# Atlantic salmon (*Salmo salar*) age at maturity is strongly affected by temperature, population and age-at-maturity genotype

**DOI:** 10.1093/conphys/coac086

**Published:** 2023-01-23

**Authors:** Eirik R Åsheim, Paul V Debes, Andrew House, Petra Liljeström, Petri T Niemelä, Jukka P Siren, Jaakko Erkinaro, Craig R Primmer

**Affiliations:** Organismal and Evolutionary Biology Research Programme, Faculty of Biological and Environmental Sciences, University of Helsinki, 00014 Helsinki, Finland; Institute of Biotechnology, Helsinki Institute of Life Science (HiLIFE), University of Helsinki, Helsinki 00014, Finland; Lammi Biological Station, Faculty of Biological and Environmental Sciences, University of Helsinki, 16900 Lammi, Finland; Organismal and Evolutionary Biology Research Programme, Faculty of Biological and Environmental Sciences, University of Helsinki, 00014 Helsinki, Finland; Department of Aquaculture and Fish Biology, Hólar University, Sauðárkrókur 550, Iceland; Organismal and Evolutionary Biology Research Programme, Faculty of Biological and Environmental Sciences, University of Helsinki, 00014 Helsinki, Finland; Institute of Biotechnology, Helsinki Institute of Life Science (HiLIFE), University of Helsinki, Helsinki 00014, Finland; Lammi Biological Station, Faculty of Biological and Environmental Sciences, University of Helsinki, 16900 Lammi, Finland; Organismal and Evolutionary Biology Research Programme, Faculty of Biological and Environmental Sciences, University of Helsinki, 00014 Helsinki, Finland; Lammi Biological Station, Faculty of Biological and Environmental Sciences, University of Helsinki, 16900 Lammi, Finland; Organismal and Evolutionary Biology Research Programme, Faculty of Biological and Environmental Sciences, University of Helsinki, 00014 Helsinki, Finland; Institute of Biotechnology, Helsinki Institute of Life Science (HiLIFE), University of Helsinki, Helsinki 00014, Finland; Natural Resources Institute Finland (LUKE), 90014 Oulu, Finland; Organismal and Evolutionary Biology Research Programme, Faculty of Biological and Environmental Sciences, University of Helsinki, 00014 Helsinki, Finland; Institute of Biotechnology, Helsinki Institute of Life Science (HiLIFE), University of Helsinki, Helsinki 00014, Finland

**Keywords:** temperature, life history, Genetics

## Abstract

Age at maturity is a key life history trait involving a trade-off between survival risk and reproductive investment, and is an important factor for population structures. In ectotherms, a warming environment may have a dramatic influence on development and life history, but this influence may differ between populations. While an increasing number of studies have examined population-dependent reactions with temperature, few have investigated this in the context of maturation timing. Atlantic salmon, a species of high conservation relevance, is a good study species for this topic as it displays considerable variation in age at maturity, of which a large proportion has been associated with a genomic region including the strong candidate gene *vgll3*. Until now, the effect of this gene in the context of different environments and populations has not been studied. Using a large-scale common-garden experiment, we find strong effects of temperature, population-of-origin, and *vgll3* genotype on maturation in 2-year-old male Atlantic salmon (*Salmo salar)*. With a temperature difference of 1.8°C, maturation probability was 4.8 times higher in the warm treatment than the cold treatment. This temperature effect was population-specific and was higher in the southern (60.48°N) compared to the northern (65.01°N) population. The early maturation vgll3*E allele was associated with a significantly higher maturation probability, but there was no *vgll3* interaction with temperature or population. Both body condition and body mass associated with maturation. The body mass association was only present in the warm treatment. Our findings demonstrate that (i) populations can vary in their response to temperature change in terms of age at maturity, (ii) high intrinsic growth could be associated with higher thermal sensitivity for life history variation and (iii) *vgll3* effects on age at maturity might be similar between populations and different thermal environments.

## Teaser

Age at maturity can vary dramatically within some species. We show that, when raised in controlled conditions, Atlantic salmon age at maturity is affected greatly by temperature as well as a recently discovered age-at-maturity gene. The influence of temperature on age at maturity differed between two populations, but the effect of the age-at-maturity gene was similar in both temperatures and populations.

## Introduction

Responses of wild animal populations to the changing climate are modulated by the phenotypic changes in individuals resulting from these changes in the environment. In this context, life history traits are of special interest as they describe the reproductive investment of organisms over their lifetime (Hutchings, 2021). Reaction norms describe the pattern of phenotypic expression of a genotype in differing environments and provide information about phenotypic plasticity and the presence of genotype × environment (GxE) interactions shaping the phenotype (Hutchings, 2011, 2021). The reaction norm between environment and life history may depend on the genetic background of the organisms, and thus animals from different populations, or animals of different key genotypes, may respond differently to environmental influences (Oomen and Hutchings, 2015). This complicates the prediction and mitigation of climate change consequences for wild populations. Furthermore, teasing apart the contributions of genetics and environment can be challenging as these two factors are often correlated in wild populations. Rearing of individuals in common, controlled conditions, that is, common garden approaches, can partly resolve this issue by observing the phenotypic differences of animals with different genetic backgrounds reared in a common environment. By combining this approach with controlled variation of several environmental factors, it is possible to build an understanding of the relative contributions of genes and environment to the phenotype, as well as the interactions between them.

Age at maturity is an important life history trait as it describes at what age an organism will start reproducing (Cole, 1954). The Atlantic salmon *Salmo salar* L. 1758 is a highly relevant species for studying life history traits such as age at maturity in the context of better understanding genetic and environmental influences. Atlantic salmon displays a considerable amount of variation in age at maturity (reviewed in Mobley *et al.*, 2021) arising from a combination of the number of years spent as a juvenile in freshwater and the number of years spent at sea before returning, often to their home river, to spawn. For example, in Atlantic salmon, the time spent at sea can vary from 0 to 5 years (Fleming, 1998; Fleming & Einum, 2011), with individuals typically doubling in size with each extra year spent at sea (Hutchings and Jones, 1998; Mobley *et al.*, 2020). Furthermore, some males never leave their home river and instead mature at a small size (down to 5 g) at the parr life stage, and so, mature individuals returning from the sea can be several thousand times larger (up to 25 kg and higher) than their mature river-bound counterparts. In recent decades, there have been conservation concerns for wild Atlantic salmon stocks due to population declines, with factors suggested to have contributed to these declines including climate change, aquaculture, illegal fishing, habitat degradation, hydropower dams and harvesting of prey species (Einum *et al.*, 2008; Chaput, 2012; ICES, 2019; Dadswell *et al.*, 2021; Lennox *et al.*, 2021; Czorlich *et al.*, 2022; Harvey *et al.*, 2022; Vollset *et al.*, 2022). Some of these factors have also been associated with life history changes in the wild stocks, with some populations experiencing a decrease in the number or proportion of early-maturing individuals (Vollset *et al.*, 2022), while others are reporting a decrease in large, late-maturing individuals (Czorlich *et al.*, 2018, 2022; Olmos *et al.*, 2019). These trends thus make the study of factors impacting Atlantic salmon life-history traits highly timely and of conservation relevance as the loss of life history diversity can make populations more vulnerable to population crashes (Schindler *et al.*, 2010).

A locus including the gene *vgll3* was earlier found to explain a large amount of variation (39%) in sea age at maturity for wild male and female Atlantic salmon (Barson *et al.*, 2015), a finding that has been replicated in both wild (Ayllon *et al.*, 2015) as well as laboratory common garden studies (Verta *et al.*, 2020; Debes *et al.*, 2021; Sinclair-Waters *et al.*, 2022). Changes in *vgll3* allele frequency have also been found to associate with a trend towards earlier maturation for Atlantic salmon in the river Teno (bordering Finland and Norway) (Czorlich *et al.*, 2018). The two alleles of *vgll3* associate with either early (E) or late (L) maturation. While other studies have provided several clues for the developmental and molecular mechanisms involved in *vgll3’s* function (Kjærner-Semb *et al.*, 2018; Kurko *et al.*, 2020; Verta *et al.*, 2020; Debes *et al.*, 2021; Pashay Ahi *et al.*, 2022), currently little is known about how this gene may interact with environmental factors like temperature and available nutrition. Furthermore, although there are some indications of differing effects of *vgll3* in differing populations (Boulding *et al.*, 2019; Mohamed *et al.*, 2019), there has not been a comparison of multiple populations in a common-garden setting. Thus, assessment of whether the effect of *vgll3* differs in differing genetic and environmental contexts can help to better understand the details of the influence of this gene and its potential role in current demographic changes of wild Atlantic salmon populations.

Temperature can have a dramatic influence on the life history traits of ectotherms (Angilletta Jr *et al.*, 2004) and is known to have significant effects on maturation age in Atlantic salmon (Baum *et al.*, 2005; Fjelldal *et al.*, 2011; Friedland *et al.*, 2009; Herbinger and Friars, 1992; Imsland *et al.*, 2014; Jonsson *et al.*, 2016; Otero *et al.*, 2011) and other ectotherm species (van der Have and de Jong, 1996). With the inherent variation in life history between Atlantic salmon populations, a key question is how this variation relates to changes in temperature, and how climate change impact might depend on the life history strategy composition of a population. While there is a growing body of literature of studies on thermal reaction norms between fish of different populations and other genetic backgrounds, looking at traits like growth, survival, metabolism and gene transcription (Hutchings, 2011; Oomen and Hutchings, 2015, 2022), few studies have investigated population differences in reaction norms between temperature and age at maturity, nor interactions with large-effect locus genotypes. Understanding the interactions between factors affecting age at maturity is also of relevance for management and conservation efforts in wild populations as well as for promoting sustainable harvest in species such as salmon (Kuparinen and Hutchings, 2017). For example, such efforts can be greatly assisted by accurate model predictions on future responses to climate change, but predictions need to be based on realistic models of how populations respond to variations in climate and how genetic parameters may interact in this process. Changes in population life history strategy composition may also be an important aspect of population responses to anthropogenic influence (Czorlich *et al.*, 2022).

In this study, we aimed to assess how important environmental (temperature and energy availability) and genetic factors (populations and life history genotypes) influence age at maturity both alone and in interaction with each other. We did this using a common garden experiment investigating maturation age in 2170 Atlantic salmon males with differing *vgll3* genotypes originating from two Atlantic salmon populations from the Baltic Sea basin. Individuals were divided between a combination of two temperature treatments with a climate-change relevant 1.8°C temperature difference, and two feed treatments differing in nutrient proportions of lipids and caloric content. We tested (i) whether the effect of *vgll3* genotype on age at maturity differs between the populations, temperatures and feed treatments; (ii) if the effect of temperature on age at maturity is population-dependent; and (iii) if other morphological phenotypes such as body mass or condition associate with maturation, and if *vgll3* genotype could influence this relationship.

## Materials and Methods

### Study animals, crossing, initial rearing and experimental feed and temperature treatments

The parental individuals of our experimental cohorts were selected from first-generation hatchery broodstocks (Neva and Oulu, established in the 1980s), which are maintained by the Natural Resources Institute Finland (LUKE) as a part of a nationally coordinated stock supplementation scheme and for stocking obligations of hydropower companies. Such hatchery broodstocks are necessary as hydropower dams block the migratory routes of the majority of original Atlantic salmon rivers in Finland, and thus there has been little or no natural reproduction in such rivers in many decades (Erkinaro *et al.*, 2011). The Neva stock originates from the river Neva in Russia (Gulf of Finland in the North-Eastern Baltic Sea) and has been used for juvenile salmon releases in the Kymi river in South-Eastern Finland. The Oulu stock includes genetic material from several Bothnian Bay (northern Baltic Sea) rivers (mostly Skellefteå, Iijoki and Tornionjoki; the exact proportions and origins are not clearly known). In addition, some few individuals from the former, original Oulu river strain were still available and used when this mixed hatchery stock was established (Erkinaro *et al.*, 2011). Every few years, new broodstocks are created from eggs and milt stripped from mature individuals caught in the Kymi (60.48°N, 26.89°E) and Oulu (65.01°N, 25.27°E) rivers following a successful marine migration. Several thousand offspring from these crosses are maintained in government-run hatcheries until they mature, after which they are annually used to create hundreds of thousands of offspring that are stocked back into the Kymi and Oulu rivers, mostly at the smolt stage, to compensate for lost natural reproduction due to hydropower dams. The Oulu broodstock is maintained in the Taivalkoski hatchery (65.60°N, 28.04°E) and the Neva broodstock in the Laukaa hatchery, 347 km further south (62.47°N, 25.88°E). Newer broodstocks eventually replace older broodstock cohorts (they are not mixed). Although there is no intentional artificial selection in stocks maintained for wild population supplementation purposes such as these (Piironen and Heinimaa, 1998), inadvertent selection for rearing in captive conditions often occurs (e.g. Mäkinen *et al.*, 2015). However, there is also an opportunity for natural selection during the 1- to 3-year period they are in the wild during their marine migration.

To avoid crossing closely related individuals, we created a pedigree by reconstructing the parents of the broodstock individuals based on SNP genotype data (Debes *et al.*, 2020, 2021). Study individuals and their (broodstock) parents and offspring individuals were genotyped using a multiplex-PCR for 177 single nucleotide polymorphisms (SNPs) of a previously described panel (Aykanat *et al.*, 2016) as outlined in Debes *et al.* (2021). The panel included the *VGLL3*_TOP_ SNP (Barson *et al.*, 2015) that was used for designing crosses to produce offspring with specific *vgll3* genotypes (see hereafter). A subset of 131 SNPs in the panel not in high linkage disequilibrium was used for reconstructing the parents of the broodstock individuals (the grandparents of the study individuals) as outlined in Debes *et al.* (2021), and the same data were used to directly assign the parents of each study individual as described in (Debes *et al.*, 2021). This information was subsequently used to correct for individual-level relatedness in the analyses (see statistical methods).

Unrelated parents with homozygous *vgll3* genotypes were used to create a series of 2 × 2 factorials (one vgll3*EE male and female and one vgll3*LL male and female) so that each 2 × 2 factorial yielded four families, one of each of the four reciprocal *vgll3* genotypes (EE, EL, LE or LL), that is, all offspring within a family had the same *vgll3* genotype (Supplementary Material Figure S1.1-Design). For analysis, we considered the two heterozygote combinations EL and LE as one genotype, EL. Only individuals from the same population were crossed together. In total, 13 and 17 2 × 2 factorials (52 and 68 families) were created using 50 and 67 parental individuals for the Oulu and Neva populations, respectively. Eggs and milt were stripped from the parental individuals at the broodstock hatcheries in mid (Oulu) or late (Neva) October 2017, immediately transported to the Viikki campus of the University of Helsinki, Finland, and fertilizations were conducted the following day.

**Figure 1:**
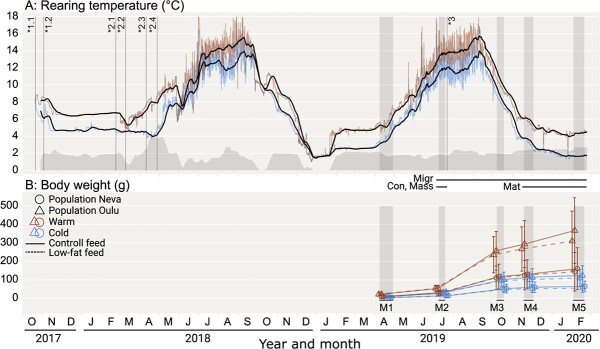
Timeline of the study showing temperatures (A) and fish growth (B). Shaded vertical areas (M1-5) indicate the timing of fish measurement sessions. The horizontal limes Con, Mass, Migr, and Mat indicate the measurement sessions from which data were taken to determine the body mass, condition, migration, and maturity status phenotypes, respectively, for use in modelling. A): Red and blue (upper and lower) lines indicate mean hourly water temperatures (of all tanks) for the warm and cold treatment, respectively. Black lines indicate the 10-day rolling water temperature average for each treatment. The grey area graph indicates the temperature difference between the 10-day rolling average of the two treatments, which averaged 1.8°C across the entire study. Periods with no temperature difference were due to heating/cooling system maintenance or technical malfunction due to very cold incoming lake water. Over the entire study, the mean temperature of the warm and cold treatment was 8.6°C and 6.9°C, respectively. Vertical lines indicate the timing of the fertilisation of Oulu (1.1) and Neva (1.2) eggs; Transport of fish to Lammi Biological Station for Oulu-Warm (2.1), Neva-Warm (2.2), Oulu-Cold (2.3), Neva-Cold (2.4) fish; and start of the feeding treatments (3). B) Points indicate mean body mass for fish in each population within each temperature-feed treatment. Points are repositioned horizontally within each measurement session to avoid overlap. Error bars indicate one standard deviation.

The fertilized eggs of each family were divided between two temperature treatments (hereafter warm, cold), following a seasonal temperature cycle but with a 2°C difference maintained between the treatments (Figure 1). The eggs were incubated as outlined in Debes *et al.* (2021). Briefly, eggs of each family were randomly and equally divided between four separate flow-through incubators, two for each temperature treatment, that is, two family replicates per temperature treatment, with families kept in separate compartments within an incubator (with randomized position). At first feeding, fish were transported to the University of Helsinki’s Lammi Biological Research Station (Lammi, Finland) and roughly equal numbers of individuals of each family from both populations were randomly chosen and placed into one of six replicate tanks of the same temperature treatment in which they had been incubated (2°C difference). Some of the fish from some factorials were used in other experiments, and for this reason, the number of Oulu fish was around double the number of Neva fish. The tank transfers took place at four different time points, due to differences in the time of first feeding caused by the different incubation temperatures and the differing fertilization times for the two populations. Respectively, the transfer dates were 23.02.2018 and 11.04.2018 for warm- and cold treatment Oulu fish, and 10.03.2018 and 24.04.2018 for the warm- and cold-treatment Neva fish (Figure 1).

The feed treatments were started in July 2019 (summer in the second-year post-fertilization) and were combined with the temperature treatments. The feed treatments were either ‘control’, in which fish received regular Raisioaqua Baltic Blend aquaculture-grade feed (17–26% fat, 18.10–20.40 kj g^−1^ depending on pellet size), or ‘low-fat’, where the feed was replaced with a custom-made fat-reduced feed of the same brand, resulting in pellets of similar size and shape to the control feed, but with lower fat content (12–13% fat, 17.25 kj g^−1^) (see Supplementary Material Table S1.1-Feed and Table S1.2-Nutrients for an overview of feed use and nutritional values). Thus, the 12 experimental tanks were divided into four treatment combinations, resulting in three tanks of each combination of temperature treatment (warm, cold) and feed treatment (control, low-fat). Tanks of different treatment combinations were evenly spread out in the research facility to minimize the occurrence of treatment-location correlations (Supplementary Material Figure S1.1-Design).

### Animal husbandry details

Following transport to Lammi Biological Station, fish were reared in experimental tanks (1.00-m tall, 2.77-m wide). The experimental tanks utilized a flow-through system of water that was pumped from a nearby lake (Pääjärvi) at ~12 m depth. To reduce pathogen load, the incoming lake water was treated with UV light before entering the tanks. Water entered the tanks through a horizontal spray bar that created a circular flow in the tanks (which was standardized between tanks), and the water level and flow rate were increased over time as the fish grew. The water temperature in the tanks followed the seasonal lake temperature curve, while a heat-exchange system aimed to maintain a 2°C difference between the warm and cold treatments (Figure 1). Lighting was automated (on/off) and set to follow the local sunrise/sunset times (at 61.05°N, 25.04°E). Over the entire study, the mean temperatures of the warm and cold treatments were 8.6°C and 6.9°C, respectively. The mean realized temperature difference was slightly lower (1.8°C) than the targeted 2°C difference due to heating/cooling system maintenance or technical malfunction due to very cold incoming lake water resulting in several short periods with no temperature difference between tanks during the first 15 months of the experiment (Figure 1).

Fish were fed *ad libitum* throughout daylight hours with body-size-matched pellets (Supplementary Material Table S1.1-Feed, Table S1.2-Nutrients) of commercial fish feed (Hercules, Raisioaqua, Raisio, Finland). Feeding was conducted manually for the first 3–4 months (until mid-June 2018), after which, an automated feed delivery system was used (Arvo-Tek Oy, Finland). To adjust feed sizes and amounts in the early phase, a subsample of fish (120–300) was measured in July, August, October and the end of November 2018. After that, feed amounts and sizes were adjusted based on size data from the regular phenotypic measurements which started in April 2019 (described below). Internal tank surfaces were scrubbed clean at least once per week. Tanks were visually inspected on a daily basis; dead fish were removed from the tanks, and any moribund fish were removed and euthanized with an MS222 overdose (0.250 g L^−1^, sodium bicarbonate-buffered). To provide the fish with environmental enrichment, half of each tank was covered with a dark-green camouflage mesh. These covers were installed at the end of April 2018 for the warm tanks, and in the middle of June 2018 for the cold tanks so that fish had experienced similar degree-days when the nets were installed.

### Weighing, measurements and maturation checks

At the first measurement in April 2019 (Figure 1; M1), all individuals were tagged with a passive integrated transponder (PIT-tag) inserted into the abdominal cavity about half a centimetre caudally from the right-side pectoral fin using sterilized needles. At the same time, a small fin clip was taken from their caudal fin, allowing for genotyping, sex determination and parental assignment as in Debes *et al.* (2021), and thereby, individual identification from that point on.

Individual phenotypic characteristics were recorded five times between April 2019 and February 2020 at 2- to 3-month intervals (Figure 1; M1-M5). These include body mass, body length and two life history phenotypes. These phenotypes were migration phenotype (smolt or parr; which in wild fish indicates the initiation of marine migration) and status of sexual maturity. Migration phenotype was checked at every measurement session from June 2019 to February 2020 (M2-M5). Maturation status was checked in November/December 2019 and February 2020 (M4-M5). Maturation status was checked by carefully stroking each individual’s abdomen towards the vent. Fish releasing milt were categorized as mature. No females showed any signs of maturation, for example, bloated belly. Migrant (smolt) versus resident (parr) phenotype was checked using criteria including level of silvering and occurrence of parr marks. Individuals were recorded as having smolted from the time point following the last recording of resident (parr) characteristics.

For measurements, fish were netted from their holding tank to a continuously aerated anaesthetic bath (MS222, 0.125 g L^−1^, sodium bicarbonate-buffered) at a similar temperature (within 1°C) to the tank water. Each individual’s body mass was then recorded to the nearest 0.01 g (April and July) and subsequently 0.1 g using a digital scale (Scout STX222 or STX6201, Ohaus, Parsippany, USA). Fork length (length from snout to fork of tail) was measured to the nearest mm using a digital fish-measurement board (DCS5, Big Fin Scientific, Austin, TX, USA), after which migration and maturation phenotypes were recorded and the fish were returned to its tank. Those performing the measurements were blind to the genotype and population of origin of the fish, but not temperature and feeding treatment.

## Statistical Analysis

### Sample size

As no females matured during the focal period, this study focuses solely on males. Due to the initially unknown rates of early maturation and mortality, we aimed for a sample size as high as possible given our supply of eggs from the hatcheries. This was to ensure we would have sufficient statistical power to test for the direct- and interaction-effects of our genetic and environmental factors. By April 2019, a total of 2657 males were tagged. By the following winter (February 2020), 263 males died prematurely, while 124 had been euthanized for use in another project (balanced amongst tanks, sex, *vgll3* genotypes and families). A further 98 males were excluded due to incomplete genotype data, and two were excluded due to other incomplete data. A total of 2170 males were thus included in the final analysis.

### Dataset and included variables

Each male individual counted as one observation. Pedigree data were included in all the models to account for family structure and relatedness (see modelling approach below). We included only fish with successfully determined *vgll3* genotypes, parental identities and sex. The variables included were *vgll3* genotype (EE, EL, LL), population of origin (Neva, Oulu), feeding treatment (control, low-fat), temperature treatment (cold, warm), migration phenotype status as observed by February 2020 (migrant, resident), maturation status by February 2020 (matured, not matured), log body mass (g, mean centered and SD scaled) and body condition (%, mean centered and SD scaled) in July 2019. This timepoint for body condition and mass was chosen as the one most likely relevant for future maturation, representing the state of body reserves before the enlarging gonads start influencing body condition (Rowe *et al.*, 1991). Body condition was calculated as the residuals of a linear model of the log body mass (g) against the log body length (mm) on the entire study population, thus being represented as percent difference in body mass from the expected body mass (given length). *vgll3* genotype was split into two variables, one for the gene’s additive effect (vgll3_add_, coded EE = 1, EL = 0, LL = -1) and one for the dominance-effect, i.e. deviance from an additive pattern (vgll3_dom_, coded EE = 0, EL = 1, LL = 0) as in Xiang *et al.* (2018).

### Modelling approach for maturation probability

We used a general linear mixed-effect modelling approach (Bernoulli distributed, logit link) to examine how maturation probability (response variable) associated with the explanatory variables *vgll3* genotype, population of origin, feed treatment, temperature treatment, body condition, body mass and migration phenotype. Although the covariates body condition, body mass and migration phenotype were not experimentally manipulated variables, they were included in the full model as explanatory variables to improve the model’s overall fit and to examine how these biologically relevant covariates interact with the genetic and environmental factors (Model-Mat-Cov or ‘full model’). For comparison, we also fitted an alternative no-covariate model which excluded body condition, body mass, and migration phenotype (Model-Mat-Nocov or ‘no-covariate model’).

To test if the effect of *vgll3* differed between populations, temperature treatments or feed treatments, we fitted interactions between *vgll3* and temperature treatment, population of origin, and feeding treatment. Additionally, in the full model, to test if the effect of *vgll3* associated with any of the phenotypic covariates, we also fitted interactions between *vgll3* and body condition, body mass, and migration phenotype.

To test if the effect of temperature is population dependent, we fitted interactions between population and temperature. To account for the possibility that the effect of the phenotypic covariates could differ between temperatures and populations, we fitted temperature and population interactions with the covariates body condition, body mass and migration phenotype.

Rearing tanks were included as random effects (on the intercept) to account for between-tank (i.e. environmental) variation. Relatedness and family structure was accounted for by including the pedigree information (up to the grandparents) into the model using an animal model approach (Henderson, 1973; Wilson *et al.*, 2010), that is, using the inverse of the additive genetic relatedness matrix (generated from the pedigree) to fit an effect of the individual animal (−and its relation to other individuals) as a random effect (on the intercept), which also gives an estimate of the additive genetic variance. This is preferable to adding family ID as a random effect, as it enables accounting for differing levels of relatedness such as full- and half-sibs. Heritability was calculated using the no-covariate model only, using the estimates of additive genetic variance as described in de Villemereuil (2021). Variance explained by *vgll3* was estimated as in Debes *et al.* (2021).

### Supplemental covariate models

Four supplemental models were fitted to explore whether *vgll3* associated with any of the three non-independent covariates as response variables: body mass (Model-Mass), body condition (Model-Cond), and migration phenotype (Model-MigPheno). These models were fitted using the same explanatory variables as the no-covariate maturation probability model (*vgll3*, temperature, population, feed), but with the following differences: Model-Mass and Model-Cond were fitted using an identity-link instead of a logit link, making them linear mixed-effect models instead of generalized linear mixed-effect models; in addition, Model-Mass and Model-Cond did not include feeding treatment as an explanatory variable since the measure of body mass and condition used in these models was recorded before the feeding treatment started (Figure 1). Finally, to allow for a closer examination of *vgll3*’s effect on body condition, an additional model was fitted for body condition including migration phenotype as a covariate (Model-Cond-Cov).

### Technical approach to model fitting

All models were fitted and analysed using a Bayesian approach for generalized and non-generalized linear mixed models. Models were fitted using *Rstan* via the *R* package *brms*. All models were fitted using 4 MCMC chains run for 3000 transitions, discarding the 500 first transitions of each chain for warmup, thus totalling 10 000 posterior samples for each model. For the maturation- and migration phenotype models, prior distributions of the intercept, effect sizes and the SDs of the random effects were all set to a relatively non-informative normal distribution with a mean of 0 and a standard deviation of 2. For the body condition and body mass models, the same parameters were given priors with a normal distribution of 0 and a standard deviation of 1. All model fits were verified using a visual posterior predictive check and checked for influential points by inspecting pareto k diagnostic values. Model-Mass had a large proportion of highly influential points (23.1% of values with K > 0.7), motivating a more careful interpretation of this model. Full model summaries can be found in Supplementary Material S2. Interactions were generally considered non-significant when the 95% credible interval of their effect size included 0. For some of those cases (noted in results), we simplified the model estimates by calculating the unconditional (marginal) mean estimates of the main effects. Unconditional estimates were calculated as the mean of one effect (i.e. *vgll3*) over all levels of the non-significant interaction variable (i.e feeding treatment). All these calculations were done using the posterior distributions of the parameter estimates (effect sizes) taken from the rstan output.

### Statistical software

All analyses were performed in the Rstudio v.2022.02.3 (RStudio Team, 2022) software environment running R v.4.1.2 (R Core Team, 2021) and Rstan v2.21.5 (Stan Development Team, 2022). R packages used for analysis were brms v.2.17.0 (Bürkner, 2017, 2018, 2021) for working with Rstan models, loo v.2.5.1 (Vehtari *et al.*, 2017, 2022) for inspecting pareto k diagnostic values, ggplot2 v3.3.5 (Wickham *et al.*, 2021) for visualization and tidyverse v1.3.1 (Wickham *et al.*, 2019) for various programming and data management tasks.

## Results

### Observed maturation rates

The overall male maturation rates in the Oulu and Neva populations were 13.3% and 32.6%, respectively (of 1335 and 835). Across-population maturation rates in the cold and warm treatments were 6.7% and 36.6% (of 1154 and 1016), respectively; while *vgll3* genotype-specific maturation rates were 6.6%, 18.2% and 35.6% for *vgll3* genotypes LL, EL and EE, respectively (of 457, 1095 and 618), respectively (Table 1).

**Table 1:** Observed maturation rates.

Group	LL	EL	EE	Total
Neva	13.6% (147)	28.7% (422)	49.2% (266)	32.6% (835)
Oulu	3.2% (310)	11.6% (673)	25.3% (352)	13.3% (1335)
Warm	13.0% (215)	32.9% (517)	61.3% (284)	36.6% (1016)
Cold	0.8% (242)	5.0% (578)	13.8% (334)	6.7% (1154)
Neva, warm	27.4% (73)	48.8% (217)	76.4% (140)	54.2% (430)
Neva, cold	0.0% (74)	7.3% (205)	19.0% (126)	9.6% (405)
Oulu, warm	5.6% (142)	21.3% (300)	46.5% (144)	23.7% (586)
Oulu, cold	1.2% (168)	3.8% (373)	10.6% (208)	5.1% (749)
Total	6.6% (457)	18.2% (1095)	35.6% (618)	20.7% (2170)

Shows observed maturation rates for male Atlantic salmon for all combinations of *vgll3* genotype, temperature treatments and population of origin. The numbers in parentheses indicate the total number of fish in that group

### Vgll3

**Figure 2:**
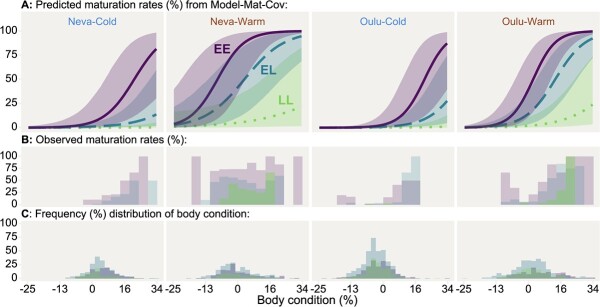
Predicted (A) maturation probabilities and observed (B) maturation rates for Atlantic salmon of three different *vgll3* genotypes (Purple-solid=EE, Blue-dashed=EL, Green-dotted=LL), two temperature treatments (Cold, Warm), and two populations of origin (Neva, Oulu), plotted against body condition. C) shows corresponding frequency distributions of body condition. Body condition represents the percentage difference in body mass from the expected body mass given body length. Lines represent the mean predicted maturation probability, with shaded areas around the lines indicating the 95% credible interval for the predictions (overlap is not an indicator of significance). Predictions are based on the full model for maturation probability (Model-Mat-Cov, Figure 3, Table S2.4). Body mass for predictions is set to the mean of the whole study population and the migration-phenotype parameter is set to 0.5 (giving an estimate unconditional on migration-phenotype).

**Figure 3:**
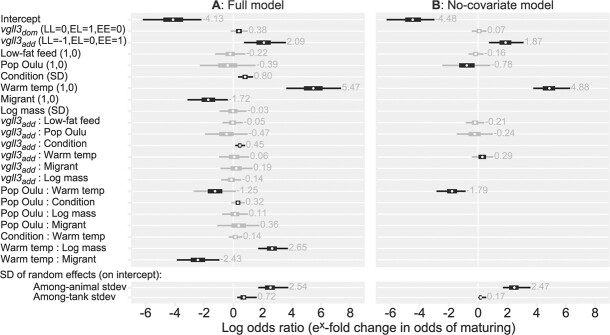
Effect sizes (parameter estimates) for the two maturation probability models. A) Parameter estimates for the full model (Model-Mat-Cov). B) A simplified model that only includes independent variables (Model-Mat-Nocov). Effects are shown as log odds ratios, thus indicating relative change in odds (odds = probability of maturing divided by the probability of not maturing); Odds 0, 1 and infinite convert to probabilities 0, 50% and 100%, respectively. Thick and thin sections of bars indicate 50% and 97.5% credible intervals, respectively. As a visual aid, intervals are coloured grey if they include 0. Grey numbers show the mean parameter estimate. Parentheses indicate the levels of the variables, and all variables are set to 0 for the intercept. The first level in parenthesis is the written level. Body condition and log body mass are SD scaled and mean-centred (mean=0), so the parameter estimates indicate the effect of increasing or decreasing either of these variables with one SD. The *vgll3*_dom_ parameter indicates the degree of dominance displayed by either of the alleles. The lower section shows the standard deviation of the random effects, representing the degree of among-tank variation and among-animal variation (additive genetic standard deviation). The full model summaries can be found in the supplementary materials (Supplementary Tables S2.4 and S2.5)

Maturation probability was higher for each carried vgll3*E allele (Table 2, Figure 2). This increase was largely additive as we found no significant dominance effect of either allele. Only in the full maturation model were there indications of a dominance effect for the E allele, but the effect was small and its 95% credible interval overlapped with zero (Model-Mat-Cov, Figure 3). The only variable having a clearly significant interaction with *vgll3* was body condition, and as such, no significant interactions with *vgll3* (on maturation probability) were found for population, temperature, feed, body mass or migration phenotype. The full maturation model (Model-Mat-Cov, Figure 3) estimated that each carried vgll3*E allele increased the odds of maturation 7.42-fold [95% CI: 3.23, 20.17] (unconditional on interactions with feed, temperature, population, and migration phenotype), and that each carried E allele was estimated to increase the body-condition effect 1.56-fold [95% CI: 1.13, 2.21]. *Vgll3* effects on the migration phenotype, body condition, and body mass covariates were all close to zero (Supplementary Material Figure S2.2, S2.3, S2.4Table S2.3, S2.4, S2.5).

**Table 2:** Predicted maturation probabilities.

Group	LL	EL	EE
Neva-Cold	0.2% [0.0%,1.5%]	1.3% [0.2%,5.1%]	7.2% [1.2%,26.3%]
Neva-warm	14.0% [2.2%,50.6%]	61.6% [30.7%,86.7%]	93.2% [75.6%,98.8%]
Oulu-Cold	0.1% [0.0%,0.8%]	0.6% [0.1%,2.0%]	2.6% [0.5%,9.5%]
Oulu-warm	1.6% [0.2%,7.1%]	10.7% [3.5%,24.4%]	43.4% [16.9%,73.9%]

Shows predicted maturation probabilities based on the no-covariate maturation model (Model-Mat-Nocov) for male Atlantic salmon in different grouped combinations of *vgll3* genotype, temperature treatment and population of origin. Brackets indicate 50% and 97.5% credible intervals. For these predictions, feed treatment was set to control-feed

### Temperature and population

**Figure 4:**
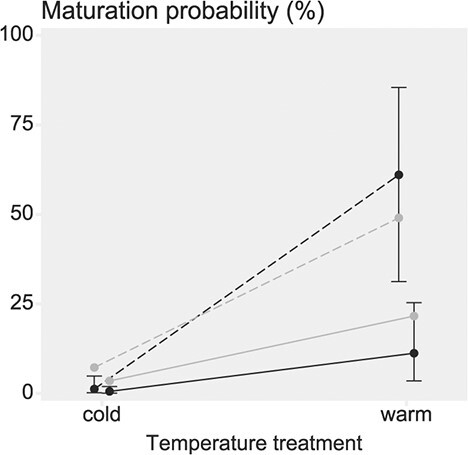
Predicted maturation probabilities (black) and observed maturation rates (grey) for Atlantic salmon from the Oulu (solid line) and Neva (dashed line) populations in each temperature treatment (mean temperatures: cold=6.9°C, warm=8.6°C) with the vgll3*EL genotype in the control-feed treatment. The predictions are based on the no-covariate maturation model (Model-Mat-Nocov, Figure 3, Supplementary Table S2.5). Error bars show the 95% credible interval of each prediction (overlap is not an indicator of significance).

Maturation probability was higher in the warm temperature treatment compared to the cold, and temperature and population interacted so that the maturation probability difference between temperatures was higher in the Neva population (Figure 4, Figure 3, Table 2). Compared to the cold-treatment fish, the warm-treatment Neva and Oulu fish were estimated to have a 131.87-fold [95% CI: 44.06, 539.21] and 20.89-fold [9.14, 56.71], respectively, higher odds of maturing (Model-Mat-Nocov, Figure 3). Maturation probability was thus higher for Neva fish, but only significantly so in the warm temperature treatment. Compared to Oulu fish, Neva fish had a 13.88-fold [95% CI: 3.08, 76.89] higher odds of maturing in the warm treatment, but only a 2.20-fold [95% CI: 0.47, 11.07] higher odds of maturing in the cold treatment (in the no-covariate model).

The estimated population effect and interaction with temperature was reduced in the full model, which included the covariates body mass, body condition and migration phenotype (Model-Mat-Cov, Figure 3); In that model (using the same comparison as above), compared to Oulu fish, Neva fish had a 4.32-fold [95% CI: 0.54, 37.30] higher odds of maturing in the warm treatment, and a 1.39-fold [95% CI: 0.19, 10.48] higher odds of maturing in the cold treatment.

Body mass was higher in the Neva population and in the warm temperature treatment, with the modelled body mass of Neva fish estimated to be 151.44% [95% CI: 95.66, 223.48] higher than Oulu fish, and the body mass of fish in the warm treatment being 73.73% [95% CI: 44.85, 107.00] higher than in the cold treatment (Model-Mass, Supplementary Material Figure S2.3). The probability of smolting (transitioning to the migrant phenotype) was higher for the Neva population and in the warm treatment, with the Neva fish having an estimated 18.69-fold [95% CI: 3.95, 104.74] increase in the odds of smolting compared to Oulu, and the warm-treatment fish having a 20.87-fold [95% CI: 5.99, 93.13] increase in the odds of smolting compared to the cold-treatment fish (Model-MigPheno, Supplementary Material Figure S2.4). There were no significant interactions between population and temperature in their effect on body mass or migration phenotype.

### Body condition

Maturation probability increased with higher body condition (Figure 2), and this effect had a small and slightly uncertain interaction with population so that the effect of body condition was slightly higher for Oulu fish (Model-Mat-Cov, Figure 3). The effect of body condition was similar in both temperatures (no significant interaction). For an Oulu fish, an increase in body condition of one standard deviation resulted in a 3.30-fold [95% CI: 2.37, 5.07] increase in the predicted odds of maturing, while for a Neva fish, the increase in predicted odds was 2.28-fold [95% CI: 1.53, 3.71] (both estimates unconditional on temperature). See Supplementary Material Table S2.1 for observed body conditions.

### Body mass

Higher body mass was associated with higher maturation probability, but only in the warm temperature treatment (Supplementary Material Figure S2.1). The effect of body mass was similar in both populations (no significant interaction). The full maturation model (Model-Mat-Cov, Figure 3) estimated that an increase in body mass by one SD would increase the odds of maturing 15.06-fold [95% CI: 7.03, 40.89] in the warm treatment and 1.00-fold [95% CI: 0.46, 2.27] in the cold treatment (both estimates unconditional on population). See Supplementary Material Table S2.3 for observed body mass.

### Migration phenotype

Observed maturation-rates were higher amongst fish that had smolted (transitioned to the migrant phenotype) prior to spawning, being 23% amongst migrants (smolt, *n* = 1586) and 13% amongst residents (parr, *n* = 584). However, after accounting for body mass and body condition, smolting prior to the spawning season was associated with a lower maturation probability, and this effect was stronger in the warm temperature treatment. The effect of migration phenotype was the same for all *vgll3* genotypes and in both populations (no significant interactions). The full maturation model (Model-Mat-Cov, Figure 3) estimated that the migrant phenotype in the cold treatment had a 0.22-fold [95% CI: 0.05, 0.86] lower odds of maturing compared to the resident phenotype, and 0.02-fold [95% CI: 0.00, 0.11] lower odds in the warm temperature treatment (both estimates unconditional on population). 73% (n = 1526) of the fish in this study had smolted by early 2020 (winter of third year post-fertilization), of which 30% (*n* = 357) also matured. See Supplementary Material Table S2.10 and S2.2 for an overview of combined smolting and maturation rates.

### Feed

The low-fat fed had no significant effect on maturation probability nor any detectable interactions with any other variables (Model-Mat-Cov, Model-Mat-Nocov, Figure 3).

### Random effects and heritability

In the full maturation model (Model-Mat-Cov, Figure 3), the estimated amongst-tank variation in maturation probability equated to an average 2.10-fold [95% CI: 1.32, 5.04] deviation from the model’s intercept, which indicates relatively minor tank-related environmental effects on maturation. The amongst-animal variation was larger, with an average deviation of the model’s intercept equating to a 15.04-fold [95% CI: 6.03, 59.59] change in odds of maturation from the mean. For example, for a mean maturation probability of 50%, the amongst-animal (additive genetic) standard deviation would equate to a range in probabilities going from 91% to 10%.

Total heritability (estimated from the no-covariate maturation model) including variation caused by *vgll3* was estimated at 0.68 [95% CI:0.54,0.82] indicating that around 68% of the remaining variation in maturation probability after accounting for the other model terms (temperature treatment, feed and population) could be ascribed to additive genetic effects, suggesting there is a proportionally large amount of additive genetic variance for male maturation. The additive variance contributed by *vgll3* was estimated at 0.13 [95% CI:0.05,0.23].

## Discussion

### Population and temperature

We found variation in population-level thermal reaction norms for early male maturation. Maturation probability was higher in the Neva population, but only significantly so in the warm temperature treatment. Both populations displayed considerably higher maturation rates in the warm- compared to the cold treatment, and this response was stronger for the Neva population. This could indicate higher thermal sensitivity of fish from the Neva population. As discussed below, our results also give indications that these population-dependent responses may be linked to the growth-rate of the fish in the respective populations.

Temperature is known to have a large influence on maturation age in salmon, although experimental studies have found mixed results regarding the direction of this effect. Some studies have observed an increase in parr maturation with increased temperature (Fjelldal *et al.*, 2011; Imsland *et al.*, 2014), some have observed reductions (Herbinger and Friars, 1992), and some have observed no change at all (Baum *et al.*, 2005). Some of these discrepancies are likely due to differences in the temperatures used and the timing of the temperature treatments. Our findings show that a life-long chronic difference in mean temperature of 1.8°C (from 6.9 to 8.6°C) can cause a large increase in the probability of early maturation for male Atlantic salmon, in our case going from an observed maturation rate of 6.7% to 36.6%. Furthermore, given the relatively high heritability of maturation probability at 0.68 (of which the *vgll3* locus contributes 0.13), we find that there is strong potential for natural selection on this trait, which could be a source of adaptive potential to factors such as climate change, and thus management strategies to promote maintenance of variation in this trait can have conservation benefits.

Adaptation to colder environments sometimes involves an increased growth rate to compensate for the slowed-down growth and metabolism that happens at lower temperatures (countergradient variation). This has been found for several species of fish (Conover and Present, 1990; Conover *et al.*, 1997; Yamahira and Conover, 2002; Yamahira *et al.*, 2007; Baumann and Conover, 2011), but the pattern is not universal (Belk *et al.*, 2005; Hutchings *et al.*, 2007; Oomen and Hutchings, 2016). The Oulu population originates ~500 km further north than the Neva population, where temperatures are generally 1–2°C colder than in the Kymi river where the Neva fish are stocked (outside of winter months; Supplementary Material Figure S3.1-river temperatures). Thus, with countergradient variation, we would have expected the Oulu fish to display an inherently higher growth rate compared to the Neva fish. Instead, we found that the Neva fish outperformed the Oulu fish in terms of growth at both temperatures (mean 8.6°C and 6.89°C). Follow-up research could include assessment of whether the Neva fish would maintain higher growth rates than the Oulu fish at even lower temperatures, or if the Oulu fish would be more robust to a further temperature decrease.

### *Vgll3* effects

In the time since the first finding of the association between *vgll3* and Atlantic salmon age at maturity (Ayllon *et al.*, 2015; Barson *et al.*, 2015), this effect of *vgll3* on maturation has been validated in common-garden studies using male Atlantic salmon in their first-year post fertilization (Verta *et al.*, 2020; Debes *et al.*, 2021 ; Sinclair-Waters *et al.*, 2022). The current study builds on these findings by showing that the effect of *vgll3* genotype on maturation also holds for male Atlantic salmon reared in common, but cooler and less controlled, thermal conditions than the previous studies, as well as showing that the relative effect of *vgll3* remains similar independent of a 1.8°C temperature difference, and independent of any potential genetic or epigenetic influences of the population of origin. Additionally, the design of this experiment allowed us to quantify the relative effect of *vgll3* on maturation compared to the effect of temperature, and we found that the effect of a single vgll3*E allele on maturation was 39% that of a 1.8°C temperature increase.

We modelled maturation probability using a threshold model (logit-link) in which we assume that maturation (a binary trait) is determined by some underlying liability trait that must reach a certain threshold to initiate the maturation process. The positive effect of *vgll3* genotype and temperature on the liability of maturation can be interpreted as these predictors either (i) having a positive effect on the unknown liability trait itself or (ii) lowering the liability trait’s threshold to induce maturation. Somatic growth is often assumed to be a key liability trait for maturation (Taranger *et al.*, 2010); however, by separately modelling body condition and body mass as response variables, we found *vgll3* to not significantly affect either of these traits directly, which means either that *vgll3* influence arises through other non-measured liability traits that do not perfectly associate with body condition or growth, or that *vgll3* works by changing the threshold of maturation for body condition and growth. This contrasts slightly with what was found in Debes *et al.* (2021), where a small effect of *vgll3* was found on body condition. However, that study had higher sample sizes (*n* = 2608) of both sexes from a single population (Neva) and temperature, used fish in their first-year post-fertilization (i.e. no individuals had undergone smoltification), and covered multiple timepoints reaching well into autumn. It was also noted that the presence and magnitude of the association varied across time, and between feeding treatments and sexes. Here, we used fish in their second-year post-fertilization and only included body condition in early July, so the different results may be attributed to any of these differing factors. Furthermore, 73% of the fish in our study also underwent the smolting process (Supplementary Material Table S2.10), which we found to affect body condition, and it is possible that an imperfect accounting of this effect could mask a *vgll3*-body condition association, or the smoltification process itself may modify the association.

The interaction between *vgll3* and body condition indicates an effect on the body condition threshold for affecting maturation, which becomes lower and narrower for each vgll3*E allele in the genotype (Figure 3A). On the other hand, the lack of an interaction between *vgll3* and temperature indicates that their relative effects (relative to the contribution of the other predictors) are independent of each other. The effect of *vgll3* was also similar between the two populations, indicating that any genetic or epigenetic factors that were different between the two populations did not significantly interact with the mechanisms of this large-effect locus.

We found no signs of a dominance pattern for the effect of *vgll3* on maturation probability, which is in line with what has been found in some other common-garden studies on early maturation in male Atlantic salmon (Debes *et al.*, 2021; Sinclair-Waters *et al.*, 2022), but not all (Verta *et al.*, 2020) and not with the initial GWAS study on wild-caught individuals which found a sex-dependent dominance pattern for *vgll3* (Barson *et al.*, 2015).

### Feed, body mass and condition

Acquisition of sufficient energy stores is a key part of the process leading up to sexual maturation (Berglund, 1992; Norrgård *et al.*, 2014; Rowe *et al.*, 1991; reviewed in Mobley *et al.*, 2021). In line with this, we found body mass and body condition to strongly associate with maturation probability, with individuals having larger body mass or body condition being associated with an increased probability of maturation. The observation that both body condition and mass were estimated to have significant effects within the same model could indicate that these traits have independent effects on maturation and that both traits need to be considered together to gain a complete understanding of the maturation processes. In terms of the relative effects of these traits, body mass had the strongest effect on maturation probability, and one standard deviation of log body mass had a 4.4 times larger effect than one standard deviation of body condition (in the warm temperature treatment, see below). Body condition is known to correlate with body fat content in Atlantic salmon (Herbinger and Friars, 1991) and other fish (Chellappa *et al.*, 1995; Mozsár *et al.*, 2015), and has been shown to be important for early male maturation in Atlantic salmon (Rowe *et al.*, 1991; Simpson, 1992) and in chinook salmon (*Oncorhynchus tshawytscha)* (Shearer and Swanson, 2000). The observed effect of body condition on maturation is then likely due to the influence of lipid stores (Parker and Cheung, 2020), while body mass might reflect other unrelated growth- or development-related factors.

Similar to what has been previously demonstrated in nine-spined stickleback (Kuparinen *et al.*, 2011), we found temperature to affect maturation not only through growth in body size but also through some other unknown pathway (as temperature still had a large effect on maturation after accounting for growth), further indicating that there are other biological processes important for maturation that do not involve body mass or body condition.

The effect of body mass on maturation was only observed in the warm temperature treatment, further emphasizing the differences in the influences of body mass and body condition. In the cold treatment, no effect of body mass was found, yet the effect of body condition remained. This could indicate that body mass does not start to influence maturation before the individual has reached a certain developmental threshold which is affected by temperature. As far as we are aware, such an interaction between temperature and body mass has not been described before. For future research, it might be helpful to see this finding replicated using a higher number of temperature treatments and with smaller temperature increments. If this is a general pattern between temperature, growth and maturation, it could mean that the sensitivity of different populations to climate change in terms of life history could be closely connected to their somatic growth rate.

No effect of the feeding treatment was detected for the probability of maturation, so fish that were fed the fat-reduced diet from July and onwards, that is, 4 months prior to spawning, had the same probability of maturing as those fed the control feed. This suggests that either the reduction in fat was not sufficient, and/or the maturation process had been initiated prior to the start of the treatment. This is in line with the findings reported by Debes *et al.* (2021) who applied a 2-day versus 7-day per week *ad libitum* feeding restriction treatment for a 6-week period starting in September (2–3months prior to spawning), but did not find any difference in maturation probability between the treatments despite large effects of the feeding treatment on growth and condition.

### Caveats

The controlled experimental setup we used allowed us to better understand the relative contributions of, and interactions between, environmental and genetic influences on life history phenotypes. However, the experimental nature of this study also necessitates careful consideration for extrapolating our results to wild-living populations.

Fish in this study were fed *ad libitum* rations, which while intended to reduce variation and stress caused by competition for feeding, also results in much higher growth rates than what can be expected in the wild, where diets are far more restricted (Armstrong and Schindler, 2011). This could affect the absolute rates of >2-year maturation observed in this study. As such, our conclusions mainly focus on the relative effects of the included factors (both genetic and environmental) rather than the absolute effects.

Furthermore, as our study individuals were second generation hatchery stock, there could also be some effects of unintentional domestication selection in our experimental cohort (Mäkinen *et al.*, 2015). The parents of the broodstock fish which our experimental cohort’s parents were selected from (i.e. our experimental cohorts’ grandparents) had completed a marine migration after release so there is also a 1- to 3-year period where there is an opportunity for natural selection. Additionally, there is selection against precocious parr maturation, as no such individuals are used for broodstock creation. Despite this, we still observed high rates of early maturation in our experimental cohort, in range of what has been observed in wild populations, (Baum *et al.*, 2004; Bohlin *et al.*, 1990; Heinimaa and Erkinaro, 2004; Myers and Hutchings, 1986, p. 198).

For the majority of the experiment, the temperature treatments were held at a constant difference of 2°C and, most of the time, were well below the 16°C optimum, which is often observed for Atlantic salmon (Elliott and Elliott, 2010). Thus, the warm treatment was always closer to the thermal optimum for growth than the cold treatment. Climate change effects, on the other hand, are unlikely to be this uniform throughout the seasons. For example, projections for the Baltic Sea over the next 100 years predict greater temperature increases in the summer compared to winter. If summer temperatures go beyond the optimal temperatures for growth, while winter temperatures stay below, predicting the effects of warming on Atlantic salmon growth and life history is going to be more challenging.

### Implications for conservation and management

Keeping the above limitations in mind, a key takeaway from this study of relevance for conservation and management is that a 2°C temperature difference can have large impacts on Atlantic salmon growth rate and life history development and that these impacts may differ between populations. There is also a possibility that this relationship is somehow related to the inherent population-specific growth. For future stock supplementation efforts, our results indicate that fish from different source populations may respond differently depending on the thermal environment of the hatchery they are maintained in and/or the river they are stocked into. For researchers modelling responses of Atlantic salmon populations to climate change, our results show they will need to take into account population-specific responses to thermal environments.

## Conclusion

Temperature, population of origin, and *vgll3* genotype each had a significant influence on maturation in two year old Atlantic salmon males. We found a population-dependent thermal reaction norm for maturation probability, suggesting that the two populations might respond differently to climate changes in terms of life history strategies and thus supporting conservation and management actions to preserve this life history strategy variation. We also found the effect of body mass on maturation to be highly temperature dependent, which suggests that responses to temperature changes in different populations could be connected to individual growth rates within each population. Further understanding of this pattern could be gained by exploring a larger range of temperatures. The relative influence of *vgll3* on maturation probability was the same for both temperatures and populations, suggesting that the relative contribution of *vgll3* in the maturation process is similar between these Atlantic salmon populations. We did not detect significant influences of *vgll3* on body mass or body condition, suggesting that a significant portion of *vgll3’s* influence is coming from pathways other than growth, or through lowering the growth threshold of maturation.

## Funding

This study was supported by the Academy of Finland [grant numbers 314254, 314255, 327255] and the European Articles Union’s Horizon 2020 research and innovation program [grant number 742312].

## Author contributions

Conceptualization: CRP, PVD; Data curation: ERÅ, CRP, JPS; Formal Analysis: ERÅ, JPS, PTN; Funding acquisition: CRP; Investigation: ERÅ, CRP, PVD, AH, PL, PTN; Methodology: CRP, PVD, ERÅ, AH, JPS, PL; Project administration: CRP; Resources: CRP, JE; Software: JPS, ERÅ, PTN; Supervision: CRP; Visualization: ERÅ; Writing—original draft: ERÅ; Writing—review & editing: ERÅ, CRP, JE, AH, PVD, PTN, JPS.

## Data availability

Upon peer-reviewed publication, the full datasets and the R scripts used for analysis will be made available in Zenodo via the following URL: https://www.doi.org/10.5281/zenodo.6883978

## Animal experimentation permit

The experiment was conducted under an animal experiment permit granted by the Finnish Project Authorization Board (permit ESAVI/2778/2018).

## Supplementary Material

Web_Material_coac086

## References

[ref1] Angilletta MJ Jr , Steury TD, Sears MW (2004) Temperature, growth rate, and body size in Ectotherms: fitting pieces of a life-history Puzzle1. Integr Compar Biol 44: 498–509. 10.1093/icb/44.6.498. 21676736

[ref2] Armstrong JB , Schindler DE (2011) Excess digestive capacity in predators reflects a life of feast and famine. Nature 476: 84–87. 10.1038/nature10240. 21734659

[ref3] Aykanat T , Lindqvist M, Pritchard VL, Primmer CR (2016) From population genomics to conservation and management: a workflow for targeted analysis of markers identified using genome-wide approaches in Atlantic salmon Salmo salar. J Fish Biol 89: 2658–2679. 10.1111/jfb.13149. 27709620

[ref4] Ayllon F , Kjærner-Semb E, Furmanek T, Wennevik V, Solberg MF, Dahle G, Taranger GL, Glover KA, Almén MS, Rubin CJ et al. (2015) The *vgll3* locus controls age at maturity in wild and domesticated Atlantic Salmon (Salmo salar L.) males. PLoS Genet 11: 1–15. 10.1371/journal.pgen.1005628. PMC4638356 26551894

[ref5] Barson NJ , Aykanat T, Hindar K, Baranski M, Bolstad GH, Fiske P, Jacq C, Jensen AJ, Johnston SE, Karlsson S et al. (2015) Sex-dependent dominance at a single locus maintains variation in age at maturity in salmon. Nature 528: 405–408. 10.1038/nature16062. 26536110

[ref6] Baum D , Laughton R, Armstrong JD, Metcalfe NB (2004) Altitudinal variation in the relationship between growth and maturation rate in salmon parr. J Animal Ecol 73: 253–260. 10.1111/j.0021-8790.2004.00803.x.

[ref7] Baum D , Laughton R, Armstrong JD, Metcalfe NB (2005) The effect of temperature on growth and early maturation in a wild population of Atlantic salmon parr. J Fish Biol 67: 1370–1380. 10.1111/j.0022-1112.2005.00832.x.

[ref8] Baumann H , Conover DO (2011) Adaptation to climate change: contrasting patterns of thermal-reaction-norm evolution in Pacific versus Atlantic silversides. Proc Royal Soc B 278: 2265–2273. 10.1098/rspb.2010.2479. PMC3119017 21208956

[ref9] Belk MC , Johnson JB, Wilson KW, Smith ME, Houston DD (2005) Variation in intrinsic individual growth rate among populations of leatherside chub (Snyderichthys copei Jordan & Gilbert): adaptation to temperature or length of growing season? Ecol Freshw Fish 14: 177–184. 10.1111/j.1600-0633.2005.00091.x.

[ref10] Berglund I (1992) Growth and early sexual maturation in Baltic salmon (Salmo salar) parr. Can J Zool 70: 205–211. 10.1139/z92-032.

[ref11] Bohlin T , Dellefors C, Faremo U (1990) Large or small at maturity—theories on the choice of alternative male strategies in anadromous salmonids. Annales Zoologici Fennici 27: 139–147.

[ref12] Boulding EG , Ang KP, Elliott JAK, Powell F, Schaeffer LR (2019) Differences in genetic architecture between continents at a major locus previously associated with sea age at sexual maturity in European Atlantic salmon. Aquaculture 500: 670–678. 10.1016/j.aquaculture.2018.09.025.

[ref13] Bürkner P-C (2017) Brms: an R package for Bayesian multilevel models using Stan. J Stat Softw 80: 1–28. 10.18637/jss.v080.i01.

[ref14] Bürkner P-C (2018) Advanced Bayesian multilevel Modeling with the R package brms. R Journal 10: 395–411. 10.32614/RJ-2018-017.

[ref15] Bürkner P-C (2021) Bayesian item response Modeling in R withbrms and Stan. J Stat Softw 100: 1–54. 10.18637/jss.v100.i05.

[ref16] Chaput G (2012) Overview of the status of Atlantic salmon (Salmo salar) in the North Atlantic and trends in marine mortality. J Marine Sci 69: 1538–1548. 10.1093/icesjms/fss013.

[ref17] Chellappa S , Huntingford FA, Strang RHC, Thomson RY (1995) Condition factor and hepatosomatic index as estimates of energy status in male three-spined stickleback. J Fish Biol 47: 775–787. 10.1111/j.1095-8649.1995.tb06002.x.

[ref18] Cole LC (1954) The population consequences of life history phenomena. Q Rev Biol 29: 103–137. 10.1086/400074. 13177850

[ref19] Conover DO , Brown JJ, Ehtisham A (1997) Countergradient variation in growth of young striped bass (Morone saxatilis) from different latitudes 1. Can J Fish Aquat Sci 54: 2401–2409. 10.1139/f97-147.

[ref20] Conover DO , Present TMC (1990) Countergradient variation in growth rate: compensation for length of the growing season among Atlantic silversides from different latitudes. Oecologia 83: 316–324. 10.1007/BF00317554. 28313001

[ref21] Czorlich Y , Aykanat T, Erkinaro J, Orell P, Primmer CR (2018) Rapid sex-specific evolution of age at maturity is shaped by genetic architecture in Atlantic salmon. Nat Ecol Evol 2: 1800–1807. 10.1038/s41559-018-0681-5. 30275465 PMC6322654

[ref22] Czorlich Y , Aykanat T, Erkinaro J, Orell P, Primmer CR (2022) Rapid evolution in salmon life history induced by direct and indirect effects of fishing. Science 376: 420–423. 10.1126/science.abg5980. 35201899

[ref23] Dadswell M , Spares A, Reader J, McLean M, McDermott T, Samways K, Lilly J (2022) The decline and impending collapse of the Atlantic Salmon (Salmo salar) population in the North Atlantic Ocean: a review of possible causes. Rev Fish Sci Aquac 30: 215–258. 10.1080/23308249.2021.1937044.

[ref24] Debes PV , Piavchenko N, Erkinaro J, Primmer CR (2020) Genetic growth potential, rather than phenotypic size, predicts migration phenotype in Atlantic salmon. Proc Royal Soc B 287: 1–10. 10.1098/rspb.2020.0867. PMC7423655 32693717

[ref25] Debes PV , Piavchenko N, Ruokolainen A, Ovaskainen O, Moustakas-Verho JE, Parre N, Aykanat T, Erkinaro J, Primmer CR (2021) Polygenic and major-locus contributions to sexual maturation timing in Atlantic salmon. Mol Ecol 30: 4505–4519. 10.1111/mec.16062. 34228841

[ref26] Einum S , Nislow KH, Reynolds JD, Sutherland WJ (2008) Predicting population responses to restoration of breeding habitat in Atlantic salmon. J Appl Ecol 45: 930–938. 10.1111/j.1365-2664.2008.01464.x.

[ref27] Elliott JM , Elliott JA (2010) Temperature requirements of Atlantic salmon Salmo salar, brown trout Salmo trutta and Arctic charr Salvelinus alpinus: predicting the effects of climate change. J Fish Biol 77: 1793–1817. 10.1111/j.1095-8649.2010.02762.x. 21078091

[ref28] Erkinaro J , Laine A, Mäki-Petäys A, Karjalainen TP, Laajala E, Hirvonen A, Orell P, Yrjänä T (2011) Restoring migratory salmonid populations in regulated rivers in the northernmost Baltic Sea area, northern Finland – biological, technical and social challenges. J Appl Ichthyol 27: 45–52. 10.1111/j.1439-0426.2011.01851.x.

[ref29] Fjelldal PG , Hansen T, Huang T (2011) Continuous light and elevated temperature can trigger maturation both during and immediately after smoltification in male Atlantic salmon (Salmo salar). Aquaculture 321: 93–100. 10.1016/j.aquaculture.2011.08.017.

[ref30] Fleming IA (1998) Pattern and variability in the breeding system of Atlantic salmon (Salmo salar), with comparisons to other salmonids. Can J Fish Aquat Sci 55: 59–76. 10.1139/d98-009.

[ref31] Fleming IA , Einum S (2010) Reproductive ecology: a tale of two sexes. Atlantic Salmon Ecol 1: 33–65. 10.1002/9781444327755.ch2.

[ref32] Friedland KD , MacLean JC, Hansen LP, Peyronnet AJ, Karlsson L, Reddin DG, Maoiléidigh ÓN, McCarthy JL (2009) The recruitment of Atlantic salmon in Europe. J Marine Sci 66: 289–304. 10.1093/icesjms/fsn210.

[ref33] Harvey A , Skaala Ø, Borgstrøm R, Fjeldheim PT, Christine Andersen K, Rong Utne K, Askeland Johnsen I, Fiske P, Winterthun S, Knutar S et al. (2022) Time series covering up to four decades reveals major changes and drivers of marine growth and proportion of repeat spawners in an Atlantic salmon population. Ecol Evol 12: 1–13. 10.1002/ece3.8780. PMC8976282 35386868

[ref34] van der Have TM , de Jong G (1996) Adult size in Ectotherms: temperature effects on growth and differentiation. J Theor Biol 183: 329–340. 10.1006/jtbi.1996.0224.

[ref35] Heinimaa S , Erkinaro J (2004) Characteristics of mature male parr in the northernmost Atlantic salmon populations. J Fish Biol 64: 219–226. 10.1111/j.1095-8649.2004.00308.x.

[ref36] Henderson CR (1973) Sire evaluation and genetic trends. J Anim Sci 1973: 10–41. 10.1093/ansci/1973.Symposium.10.

[ref37] Herbinger CM , Friars GW (1991) Correlation between condition factor and total lipid content in Atlantic salmon, Salmo salar L., parr. Aquaculture Res 22: 527–529. 10.1111/j.1365-2109.1991.tb00766.x.

[ref38] Herbinger CM , Friars GW (1992) Effects of winter temperature and feeding regime on the rate of early maturation in Atlantic salmon (Salmo salar) male parr. Aquaculture 101: 147–162. 10.1016/0044-8486(92)90239-H.

[ref39] Hutchings JA (2011) Old wine in new bottles: reaction norms in salmonid fishes. Heredity 106: 421–437. 10.1038/hdy.2010.166. 21224878 PMC3131971

[ref40] Hutchings, J. A. (2021). A Primer of Life Histories: Ecology, Evolution, and Application (1st ed.). Oxford University Press, Oxford, New York. 10.1093/oso/9780198839873.001.0001

[ref41] Hutchings JA , Jones M (1998) Life history variation and growth rate thresholds for maturity in Atlantic salmon, Salmo salar. can J Fisheries Aquat Sci 55: 22–47. 10.1139/d98-004.

[ref42] Hutchings JA , Swain DP, Rowe S, Eddington JD, Puvanendran V, Brown JA (2007) Genetic variation in life-history reaction norms in a marine fish. Proc Royal Soc B 274: 1693–1699. 10.1098/rspb.2007.0263. PMC2493576 17490948

[ref43] ICES . (2019). NASCO Workshop for North Atlantic Salmon At-Sea Mortality. ICES Scientific Reports, Copenhagen, Denmark. 10.17895/ICES.PUB.5979

[ref44] Imsland AK , Handeland SO, Stefansson SO (2014) Photoperiod and temperature effects on growth and maturation of pre- and post-smolt Atlantic salmon. Aquaculture International 22: 1331–1345. 10.1007/s10499-014-9750-1.

[ref45] Jonsson B , Jonsson N, Albretsen J (2016) Environmental change influences the life history of salmon Salmo salar in the North Atlantic Ocean. J Fish Biol 88: 618–637. 10.1111/jfb.12854. 26725985

[ref46] Kjærner-Semb E , Ayllon F, Kleppe L, Sørhus E, Skaftnesmo K, Furmanek T, Segafredo FT, Thorsen A, Fjelldal PG, Hansen T et al. (2018) *Vgll3* and the hippo pathway are regulated in Sertoli cells upon entry and during puberty in Atlantic salmon testis. Sci Rep 8: 1912. 10.1038/s41598-018-20308-1. 29382956 PMC5789820

[ref47] Kuparinen A , Cano JM, Loehr J, Herczeg G, Gonda A, Merilä J (2011) Fish age at maturation is influenced by temperature independently of growth. Oecologia 167: 435–443. 10.1007/s00442-011-1989-x. 21479961

[ref48] Kuparinen A , Hutchings JA (2017) Genetic architecture of age at maturity can generate divergent and disruptive harvest-induced evolution. Philo Trans Royal Soc B 372: 1–8. 10.1098/rstb.2016.0035. PMC5182431 27920380

[ref49] Kurko J , Debes PV, House AH, Aykanat T, Erkinaro J, Primmer CR (2020) Transcription profiles of age-at-maturity-associated genes suggest cell fate commitment regulation as a key factor in the Atlantic Salmon maturation process. Genetics 10: 235–246. 10.1534/g3.119.400882. PMC6945027 31740454

[ref50] Lennox RJ , Alexandre CM, Almeida PR, Bailey KM, Barlaup BT, Bøe K, Breukelaar A, Erkinaro J, Forseth T, Gabrielsen S-E et al. (2021) The quest for successful Atlantic salmon restoration: perspectives, priorities, and maxims. J Marine Sci 78: 3479–3497. 10.1093/icesjms/fsab201.

[ref51] Mäkinen H , Vasemägi A, McGinnity P, Cross TF, Primmer CR (2015) Population genomic analyses of early-phase Atlantic Salmon (Salmo salar) domestication/captive breeding. Evol Appl 8: 93–107. 10.1111/eva.12230. 25667605 PMC4310584

[ref52] Mobley KB , Aykanat T, Czorlich Y, House A, Kurko J, Miettinen A, Moustakas-Verho J, Salgado A, Sinclair-Waters M, Verta J-P et al. (2021, 2021) Maturation in Atlantic salmon (Salmo salar, Salmonidae): a synthesis of ecological, genetic, and molecular processes. Rev Fish Biol Fisheries 31: 523–571. 10.1007/s11160-021-09656-w.

[ref53] Mobley KB , Granroth-Wilding H, Ellmén M, Orell P, Erkinaro J, Primmer CR (2020) Time spent in distinct life history stages has sex-specific effects on reproductive fitness in wild Atlantic salmon. Mol Ecol 29: 1173–1184. 10.1111/mec.15390. 32077545

[ref54] Mohamed AR , Verbyla KL, Al-Mamun HA, McWilliam S, Evans B, King H, Kube P, Kijas JW (2019) Polygenic and sex specific architecture for two maturation traits in farmed Atlantic salmon. BMC Genomics 20: 139. 10.1186/s12864-019-5525-4. 30770720 PMC6377724

[ref55] Mozsár A , Boros G, Sály P, Antal L, Nagy SA (2015) Relationship between Fulton’s condition factor and proximate body composition in three freshwater fish species. J Appl Ichthyol 31: 315–320. 10.1111/jai.12658.

[ref56] Myers RA , Hutchings JA (1986) Selection against parr maturation in Atlantic salmon. Aquaculture 53: 313–320. 10.1016/0044-8486(86)90362-5.

[ref57] Norrgård JR , Bergman E, Greenberg LA, Schmitz M (2014) Effects of feed quality and quantity on growth, early maturation and smolt development in hatchery-reared landlocked Atlantic salmon Salmo salar. J Fish Biol 85: 1192–1210. 10.1111/jfb.12523. 25263188

[ref58] Olmos M , Massiot-Granier F, Prévost E, Chaput G, Bradbury IR, Nevoux M, Rivot E (2019) Evidence for spatial coherence in time trends of marine life history traits of Atlantic salmon in the North Atlantic. Fish Fish 20: 322–342. 10.1111/faf.12345.

[ref59] Oomen RA , Hutchings JA (2015) Genetic variability in reaction norms in fishes. Environ Rev 23: 353–366. 10.1139/er-2014-0077.

[ref60] Oomen RA , Hutchings JA (2016) Genetic variation in plasticity of life-history traits between Atlantic cod (Gadus morhua) populations exposed to contrasting thermal regimes. Can J Zool 94: 257–264. 10.1139/cjz-2015-0186.

[ref61] Oomen RA , Hutchings JA (2022) Genomic reaction norms inform predictions of plastic and adaptive responses to climate change. J Anim Ecol 91: 1073–1087. 10.1111/1365-2656.13707. 35445402 PMC9325537

[ref62] Otero J , Jensen AJ, L’Abée-Lund JH, Stenseth NC, Storvik GO, Vøllestad LA (2011) Quantifying the ocean, freshwater and human effects on year-to-year variability of One-Sea-winter Atlantic Salmon angled in multiple Norwegian Rivers. PLOS ONE 6: 1–11. 10.1371/journal.pone.0024005. PMC3163678 21897867

[ref63] Parker CG , Cheung E (2020) Metabolic control of teleost reproduction by leptin and its complements: understanding current insights from mammals. Gen Comp Endocrinol 292: 1–9. 10.1016/j.ygcen.2020.113467. 32201232

[ref64] Pashay Ahi E , Sinclair-Waters M, Moustakas-Verho J, Jansouz S, Primmer CR (2022) Strong regulatory effects of *vgll3* genotype on reproductive axis gene expression in juvenile male Atlantic salmon. Gen Comp Endocrinol 325: 1–6. 10.1016/j.ygcen.2022.114055. 35580687

[ref65] Piironen J , Heinimaa P (1998) Preservation programs for endangered fish stocks in Finland. Action before Extinction: An International Conference on Conservation of Fish Genetic Diversity 1: 105–114.

[ref66] R Core Team . (2021). *R: A Language and Environment for Statistical Computing*. R Foundation for Statistical Computing, Vienna, Austria. https://www.R-project.org/

[ref67] Rowe DK , Thorpe JE, Shanks AM (1991) Role of fat Stores in the Maturation of male Atlantic Salmon (Salmo salar) Parr. Can J Fish Aquat Sci 48: 405–413. 10.1139/f91-052.

[ref68] RStudio Team . (2022). RStudio: Integrated Development Environment for R. RStudio, PBC, Boston, MA. http://www.rstudio.com/

[ref69] Schindler DE , Hilborn R, Chasco B, Boatright CP, Quinn TP, Rogers LA, Webster MS (2010) Population diversity and the portfolio effect in an exploited species. Nature 465: 609–612. 10.1038/nature09060. 20520713

[ref70] Shearer KD , Swanson P (2000) The effect of whole body lipid on early sexual maturation of 1+ age male Chinook salmon (Oncorhynchus tshawytscha). Aquaculture 190: 343–367. 10.1016/S0044-8486(00)00406-3.

[ref71] Simpson AL (1992) Differences in body size and lipid reserves between maturing and nonmaturing Atlantic salmon parr, Salmo salar L. Can J Zool 70: 1737–1742. 10.1139/z92-241.

[ref72] Sinclair-Waters M , Piavchenko N, Ruokolainen A, Aykanat T, Erkinaro J, Primmer CR (2022) Refining the genomic location of single nucleotide polymorphism variation affecting Atlantic salmon maturation timing at a key large-effect locus. Mol Ecol 31: 562–570. 10.1111/mec.16256. 34716945

[ref73] Stan Development Team (2022) RStan: the R interface to Stan. https://mc-stan.org/.

[ref74] Taranger GL , Carrillo M, Schulz RW, Fontaine P, Zanuy S, Felip A, Weltzien F-A, Dufour S, Karlsen Ø, Norberg B et al. (2010) Control of puberty in farmed fish. Gen Comp Endocrinol 165: 483–515. 10.1016/j.ygcen.2009.05.004. 19442666

[ref75] Vehtari A , Gabry J, Magnusson M, Yao Y, Bürkner P-C, Paananen T, Gelman A (2022) Loo: efficient leave-one-out cross-validation and WAIC for Bayesian models. https://mc-stan.org/loo/.

[ref76] Vehtari A , Gelman A, Gabry J (2017) Practical Bayesian model evaluation using leave-one-out cross-validation and WAIC. Statistics and Computing 27: 1413–1432. 10.1007/s11222-016-9696-4.

[ref77] Verta J-P , Debes PV, Piavchenko N, Ruokolainen A, Ovaskainen O, Moustakas-Verho JE, Tillanen S, Parre N, Aykanat T, Erkinaro J et al. (2020) Cis-regulatory differences in isoform expression associate with life history strategy variation in Atlantic salmon. PLoS Genet 16: 1–23. 10.1371/journal.pgen.1009055. PMC7549781 32997662

[ref78] de Villemereuil P (2021) New version of the tutorial on heritability and MCMCglmm – Pierre de Villemereuil, https://devillemereuil.legtux.org/new-version-of-the-tutorial-on-heritability-and-mcmcglmm/

[ref79] Vollset KW , Urdal K, Utne K, Thorstad EB, Sægrov H, Raunsgard A, Skagseth Ø, Lennox RJ, Østborg GM, Ugedal O et al. (2022) Ecological regime shift in the Northeast Atlantic Ocean revealed from the unprecedented reduction in marine growth of Atlantic salmon. Sci Adv 8: 1–11. 10.1126/sciadv.abk2542. PMC8896796 35245115

[ref80] Wickham H , Averick M, Bryan J, Chang W, McGowan LD, François R, Grolemund G, Hayes A, Henry L, Hester J et al. (2019) Welcome to the tidyverse. J Open Source Software 4: 1–6. 10.21105/joss.01686.

[ref81] Wickham H , Chang W, Henry L, Pedersen TL, Takahashi K, Wilke C, Woo K, Yutani H, Dunnington D, RStudio. (2021) ggplot 2: Create Elegant Data Visualisations Using the Grammar of Graphics, Springer-Verlag, New York. https://CRAN.R-project.org/package=ggplot2

[ref82] Wilson AJ , Réale D, Clements MN, Morrissey MM, Postma E, Walling CA, Kruuk LEB, Nussey DH (2010) An ecologist’s guide to the animal model. J Animal Ecol 79: 13–26. 10.1111/j.1365-2656.2009.01639.x. 20409158

[ref83] Xiang T , Christensen OF, Vitezica ZG, Legarra A (2018) Genomic model with correlation between additive and dominance effects. Genetics 209: 711–723. 10.1534/genetics.118.301015. 29743175 PMC6028252

[ref84] Yamahira K , Conover DO (2002) Intra- Vs. interspecific latitudinal variation in growth: adaptation to temperature or seasonality? Ecology 83: 1252–1262. 10.1890/0012-9658(2002)083[1252:IVILVI]2.0.CO;2.

[ref85] Yamahira K , Kawajiri M, Takeshi K, Irie T (2007) Inter- and Intrapopulation variation in thermal reaction norms for growth rate: evolution of latitudinal compensation in Ectotherms with a genetic constraint. Evolution 61: 1577–1589. 10.1111/j.1558-5646.2007.00130.x. 17598741

