# Correction to: Atlantic salmon (Salmo salar) age at maturity is strongly affected by temperature, population and age-at-maturity genotype

**DOI:** 10.1093/conphys/coad014

**Published:** 2023-03-30

*Conserv Physiol* 11(1): coac086; doi: https://10.1093/conphys/coac086

In the originally published version of this manuscript, the figures within the paper are missing their respective captions and an incorrect supplementary file was uploaded.

This error has been corrected.

