# Correction to: Atlantic salmon (*Salmo salar*) age at maturity is strongly affected by temperature, population and age-at-maturity genotype

**DOI:** 10.1093/conphys/coae016

**Published:** 2024-03-21

This is a correction to:

Eirik R Åsheim, Paul V Debes, Andrew House, Petra Liljeström, Petri T Niemelä, Jukka P Siren, Jaakko Erkinaro, Craig R Primmer, Atlantic salmon (*Salmo salar*) age at maturity is strongly affected by temperature, population and age-at-maturity genotype, Conservation Physiology, Volume 11, Issue 1, 2023, coac086, https://doi.org/10.1093/conphys/coac086
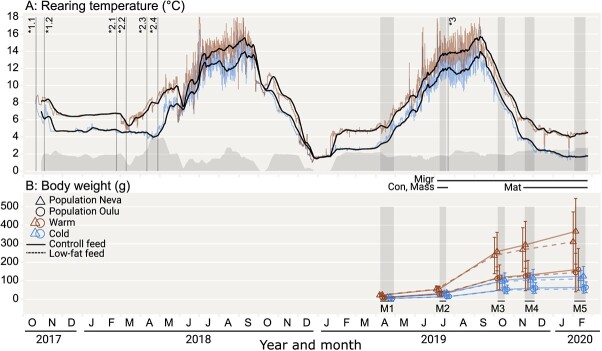


In the originally published version of this manuscript, the symbols in the legend of Figure 1 are inverted for the two populations. Population “Neva” should be represented by Triangles, and “Oulu” should be represented by Circles.

These details have been corrected only in this correction notice to preserve the published version of record.

