# Supplementary material for: Atlantic salmon (Salmo salar) age at maturity is strongly affected by temperature, population and age-at-maturity genotype
Source: Conserv Physiol. 2023 Jan 23;11(1):coac086. doi: 10.1093/conphys/coac086 (PMC9871436; doi:10.1093/conphys/coac086)
Supplement: Web_Material_coac086 [file web_material_coac086.zip › Asheim-etal-MaleMatGxE-review-suppl-revised_coac086.docx]

SUPPLEMENTARY MATERIAL 1: Supplementary methods and design


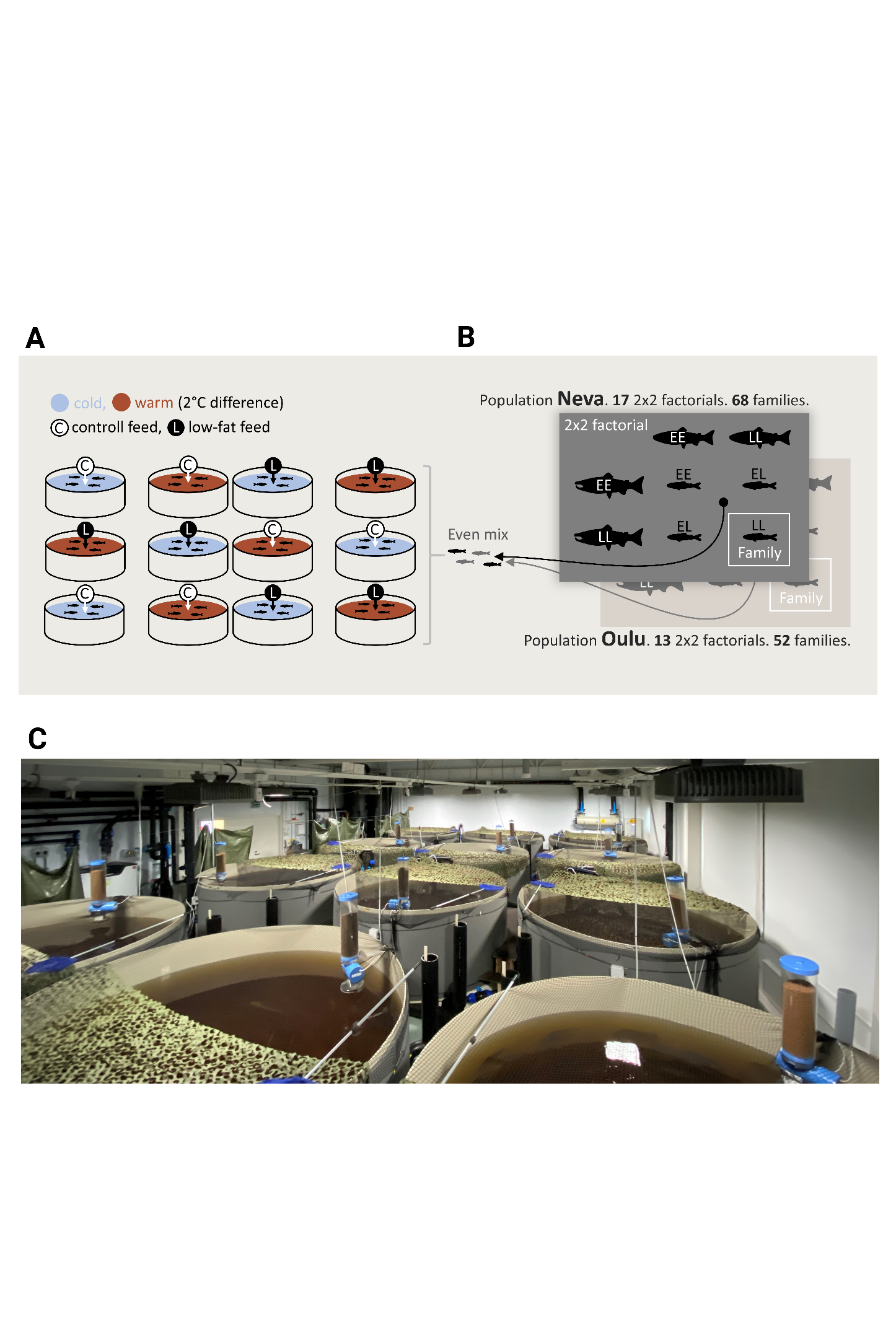


**Figure S1.1-Design**. Crossing, rearing, and experimental design. A) Shows the experimental tanks, their temperature- and feed treatments, as well as their relative positions in the room. B) Shows the crossing design, where unrelated parents from either the Neva or Oulu population were crossed in a series of 2 × 2 factorials (one *vgll3*EE* male and female and one *vgll3*LL* male and female) so that each 2 x 2 factorial yielded four families, one of each of the four reciprocal *vgll3* genotypes (EE, EL, LE or LL), i.e., all offspring within a family had the same *vgll3* genotype. Only individuals from the same population were crossed together. Roughly equal numbers of individuals from each family and population were placed into the 12 experimental tanks. C) A photograph of the experimental tanks taken in March 2020, showing tanks, feeders, and camouflage nets covering the tanks. The system relies on a constant flow of water drawn directly from the local lake Pääjärvi, which is where the water’s brown colour comes from.

**Table S1.1-Feed:** Usage overview of different pellet sizes. The table shows the date of first use of each pellet size for the different temperature- and feeding treatments. Pellet sizes were matched to the growth of the fish in the respective treatments. For future reference, this table contains dates used for the entire experiment; dates with asterisks are not included in this particular study. Feeds with end use “…” were used until the end of the experiment, and dates marked * are outside the time-scope of this paper.

| Feed | Temp | Size (mm) | Start use | End use |
| --- | --- | --- | --- | --- |
| low fat | warm | 1,2 | 10.07.2019 | *16.08.2020 |
| low fat | warm | 1,7 | 10.07.2019 | *20.07.2020 |
| low fat | warm | 2,5 | 16.08.2019 | *16.08.2020 |
| low fat | warm | 3,5 | 01.09.2019 | *15.05.2021 |
| low fat | warm | 5 | 07.10.2019 | … |
| low fat | warm | 7 | *01.05.2020 | … |
| low fat | cold | 1,2 | 17.07.2019 | *20.12.2020 |
| low fat | cold | 1,7 | 17.07.2019 | *04.04.2021 |
| low fat | cold | 2,5 | 16.08.2019 | *04.04.2021 |
| low fat | cold | 3,5 | 09.09.2019 | *04.04.2021 |
| low fat | cold | 5 | 07.10.2019 | *04.04.2021 |
| low fat | cold | 7 | *25.05.2020 | *04.04.2021 |
| controll | warm | 0,8 | 23.02.2018 | 10.07.2019 |
| Controll | warm | 1,2 | 29.05.2019 | *16.08.2020 |
| Controll | warm | 1,7 | 29.05.2019 | *20.07.2020 |
| Controll | warm | 2,5 | 16.08.2019 | *08.11.2020 |
| Controll | warm | 3,5 | 01.09.2019 | *15.05.2021 |
| Controll | warm | 5 | 07.10.2019 | … |
| Controll | warm | 7 | *01.05.2020 | … |
| controll | cold | 0,8 | 10.03.2018 | 10.07.2019 |
| Controll | cold | 1,2 | 29.05.2019 | *20.12.2020 |
| Controll | cold | 1,7 | 29.05.2019 | *04.04.2021 |
| Controll | cold | 2,5 | 16.08.2019 | *04.04.2021 |
| Controll | cold | 3,5 | 09.09.2019 | *04.04.2021 |
| Controll | cold | 5 | 07.10.2019 | *04.04.2021 |
| Controll | cold | 7 | *25.05.2020 | *04.04.2021 |

**Table S1.2-Nutrients:** Nutritional overview of different feed types and pellet sizes, as well as intended fish-size range for different pellet sizes.

| Size(mm) | Fish-size (g) | Type | % Moisture | % Ash | % Fat | % Protein | % Carbs | Energy (kj/g) |
| --- | --- | --- | --- | --- | --- | --- | --- | --- |
| 0,8 | 0.8-4 | control | 4,8 | 8,4 | 16,9 | 57,6 | 12,3 | 18,1 |
| 1,2 | 4-15 | control | 5,4 | 7,2 | 17,7 | 53 | 16,7 | 18,4 |
| 1,2 | 4-15 | low fat | 6,3 | 6,8 | 13,4 | 53,3 | 20,2 | 17,5 |
| 1,7 | 15-30 | control | 6,3 | 7,3 | 21,6 | 48,9 | 15,9 | 19,0 |
| 1,7 | 15-30 | low fat | 8,4 | 7,9 | 13,7 | 51,1 | 18,9 | 17,0 |
| 2,5 | 25-70 | control | 5,5 | 6,6 | 22,3 | 47 | 18,7 | 19,4 |
| 2,5 | 25-70 | low fat | 6,0 | 7,3 | 12,8 | 52,2 | 21,7 | 17,3 |
| 3,5 | 60-125 | control | 4,9 | 5,8 | 26,2 | 45,3 | 17,8 | 20,4 |
| 3,5 | 60-125 | low fat | 5,6 | 6,9 | 11,7 | 53,6 | 22,2 | 17,2 |
| 5 | 110-500 | control | 7,7 | 4,9 | 29,7 | 38,4 | 19,6 | 20,8 |
| 5 | 110-500 | low fat | 8,3 | 6,5 | 9,7 | 50,3 | 25,8 | 16,5 |
| 7 | 450-2300 | control | 5,9 | 4,3 | 33,7 | 35,8 | 20,0 | 22,0 |
| 7 | 450-2300 | low fat | 6,4 | 6,1 | 9,0 | 48,8 | 29,3 | 16,6 |

SUPPLEMENTARY MATERIAL 2: Supplementary results


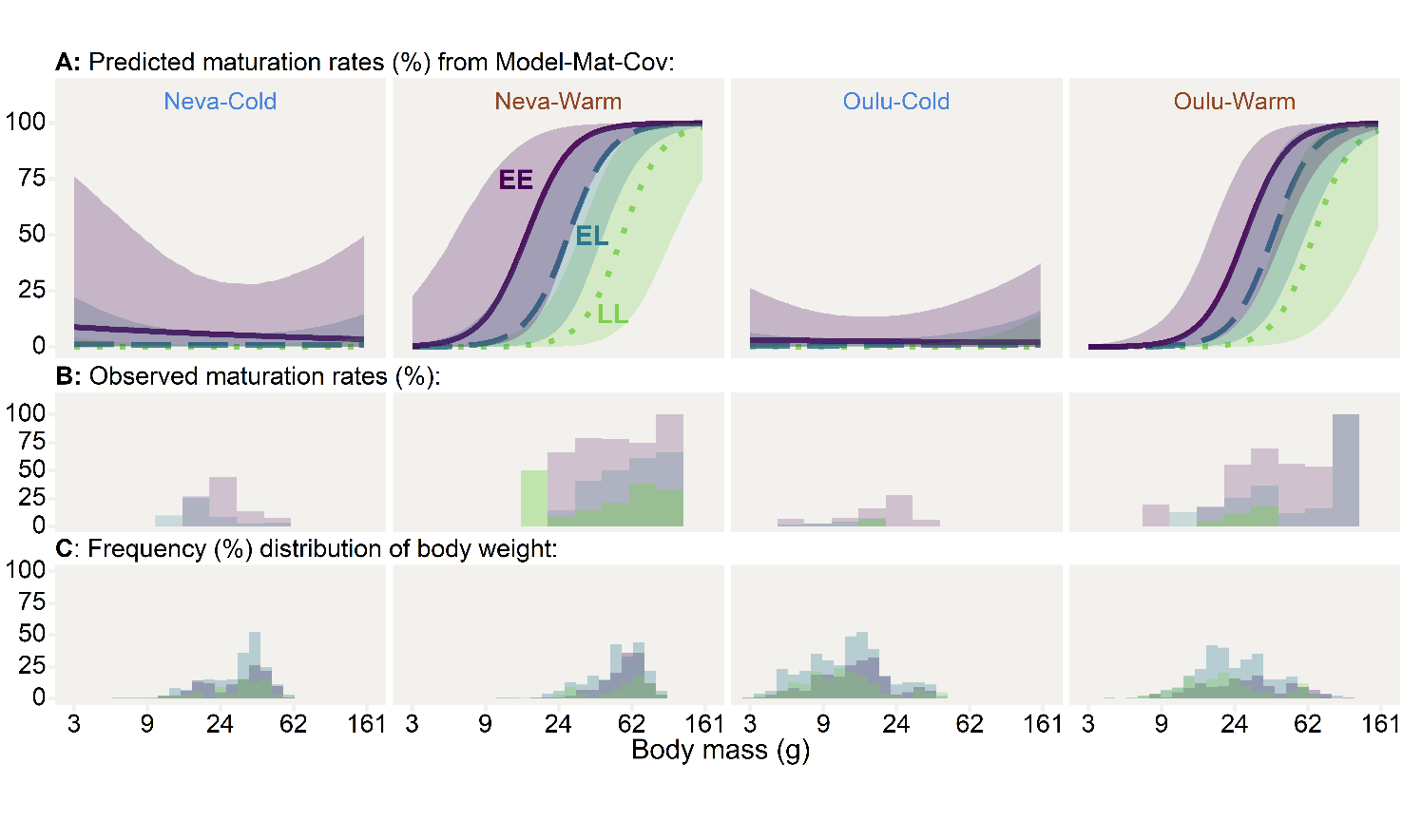


**Figure S2.1-Maturation.** Predicted maturation probabilities(A) and observed (B) maturation rates for *Salmo salar* of three different *vgll3* genotypes (Purple-solid=EE, Blue-dashed=EL, Green-dotted=LL), two temperature treatments (Cold, Warm), and two populations of origin (Neva, Oulu), plotted against log-scaled body mass together with frequency distributions of body condition (C). x-axis labels are back-transformed from log scale to represent body mass in grams. Lines represent the mean predicted maturation probability for a given body mass, with shaded areas around the lines indicating the 95% credible interval for the predictions (overlap is not an indicator of significance). Predictions are based on the full model for maturation probability (Model-Mat-Cov, Figure 3-A, Table S2.4). For the model predictions, body condition here is to the mean of the whole study population and the migration-phenotype parameter is set to 0.5 (giving an estimate lying between migrant- and resident-phenotype individuals).

**Table S2.1-CondObs.** Observed body conditions for male *Salmo salar*  in the summer of their second-year post-fertilization in different grouped combinations of *vgll3* genotype, temperature treatments and population of origin. Numbers after ± indicate SEM. Body condition is presented as percentage difference from predicted body mass (given body length). Numbers in parenthesis indicate the total number of fish in that group.

| Group | LL | EL | EE | Total |
| --- | --- | --- | --- | --- |
| Neva | 1.55%±0.54 (147) | 1.99%±0.40 (422) | 2.99%±0.48 (266) | 0.02%±0.00 (835) |
| Oulu | 0.14%±0.67 (310) | -0.59%±0.35 (673) | 1.34%±0.48 (352) | 0.00%±0.00 (1335) |
| Warm | 2.24%±0.84 (215) | 1.45%±0.41 (517) | 3.25%±0.56 (284) | 0.02%±0.00 (1016) |
| Cold | -0.87%±0.53 (242) | -0.53%±0.35 (578) | 1.03%±0.41 (334) | 0.00%±0.00 (1154) |
| Neva, warm | 0.51%±0.79 (73) | 0.10%±0.57 (217) | 0.67%±0.72 (140) | 0.00%±0.00 (430) |
| Neva, cold | 2.58%±0.73 (74) | 3.99%±0.52 (205) | 5.57%±0.54 (126) | 0.04%±0.00 (405) |
| Oulu, warm | 3.13%±1.20 (142) | 2.42%±0.56 (300) | 5.75%±0.82 (144) | 0.03%±0.00 (586) |
| Oulu, cold | -2.39%±0.66 (168) | -3.02%±0.40 (373) | -1.72%±0.48 (208) | -0.03%±0.00 (749) |
| Total | 0.59%±0.49 (457) | 0.40%±0.27 (1095) | 2.05%±0.34 (618) | 0.01%±0.00 (2170) |

**Table S2.2-SmoltObs.** Observed smolting rates for male *Salmo salar* at two years post-fertilization for different grouped combinations of *vgll3* genotype, temperature treatments and population of origin. Numbers in parenthesis indicate total number of fish in that group.

| Group | LL | EL | EE | Total |
| --- | --- | --- | --- | --- |
| Neva | 89.8% (147) | 88.6% (422) | 94% (266) | 90.5% (835) |
| Oulu | 55.8% (310) | 62.1% (673) | 67.9% (352) | 62.2% (1335) |
| Warm | 84.7% (215) | 90.9% (517) | 92.3% (284) | 90% (1016) |
| Cold | 50.8% (242) | 55.7% (578) | 68% (334) | 58.2% (1154) |
| Neva, warm | 94.5% (73) | 96.8% (217) | 98.6% (140) | 97% (430) |
| Neva, cold | 85.1% (74) | 80% (205) | 88.9% (126) | 83.7% (405) |
| Oulu, warm | 79.6% (142) | 86.7% (300) | 86.1% (144) | 84.8% (586) |
| Oulu, cold | 35.7% (168) | 42.4% (373) | 55.3% (208) | 44.5% (749) |
| Total | 66.7% (457) | 72.3% (1095) | 79.1% (618) | 73.1% (2170) |

**Table S2.3-MassObs.** Observed body mass (g) for male *Salmo salar* in the summer of their second-year post-fertilization in different grouped combinations of *vgll3* genotype, temperature treatments and population of origin. Numbers after ± indicate the SEM. Numbers in parenthesis indicate the total number of fish in that group

| Group | LL | EL | EE | Total |
| --- | --- | --- | --- | --- |
| Neva | 43.1±1.6 (147) | 42.7±0.9 (422) | 44.7±1.1 (266) | 43.4±0.6 (835) |
| Oulu | 18.6±0.8 (310) | 20.0±0.5 (673) | 22.0±0.8 (352) | 20.2±0.4 (1335) |
| Warm | 34.9±1.5 (215) | 38.2±0.9 (517) | 44.2±1.2 (284) | 39.2±0.7 (1016) |
| Cold | 19.0±0.8 (242) | 20.4±0.5 (578) | 21.1±0.7 (334) | 20.3±0.4 (1154) |
| Neva, warm | 55.1±2.3 (73) | 53.3±1.2 (217) | 56.5±1.2 (140) | 54.7±0.8 (430) |
| Neva, cold | 31.3±1.2 (74) | 31.5±0.8 (205) | 31.5±1.0 (126) | 31.5±0.5 (405) |
| Oulu, warm | 24.5±1.3 (142) | 27.2±0.8 (300) | 32.2±1.5 (144) | 27.8±0.7 (586) |
| Oulu, cold | 13.6±0.7 (168) | 14.3±0.4 (373) | 14.9±0.5 (208) | 14.3±0.3 (749) |
| Total | 26.5±0.9 (457) | 28.8±0.6 (1095) | 31.7±0.8 (618) | 29.1±0.4 (2170) |


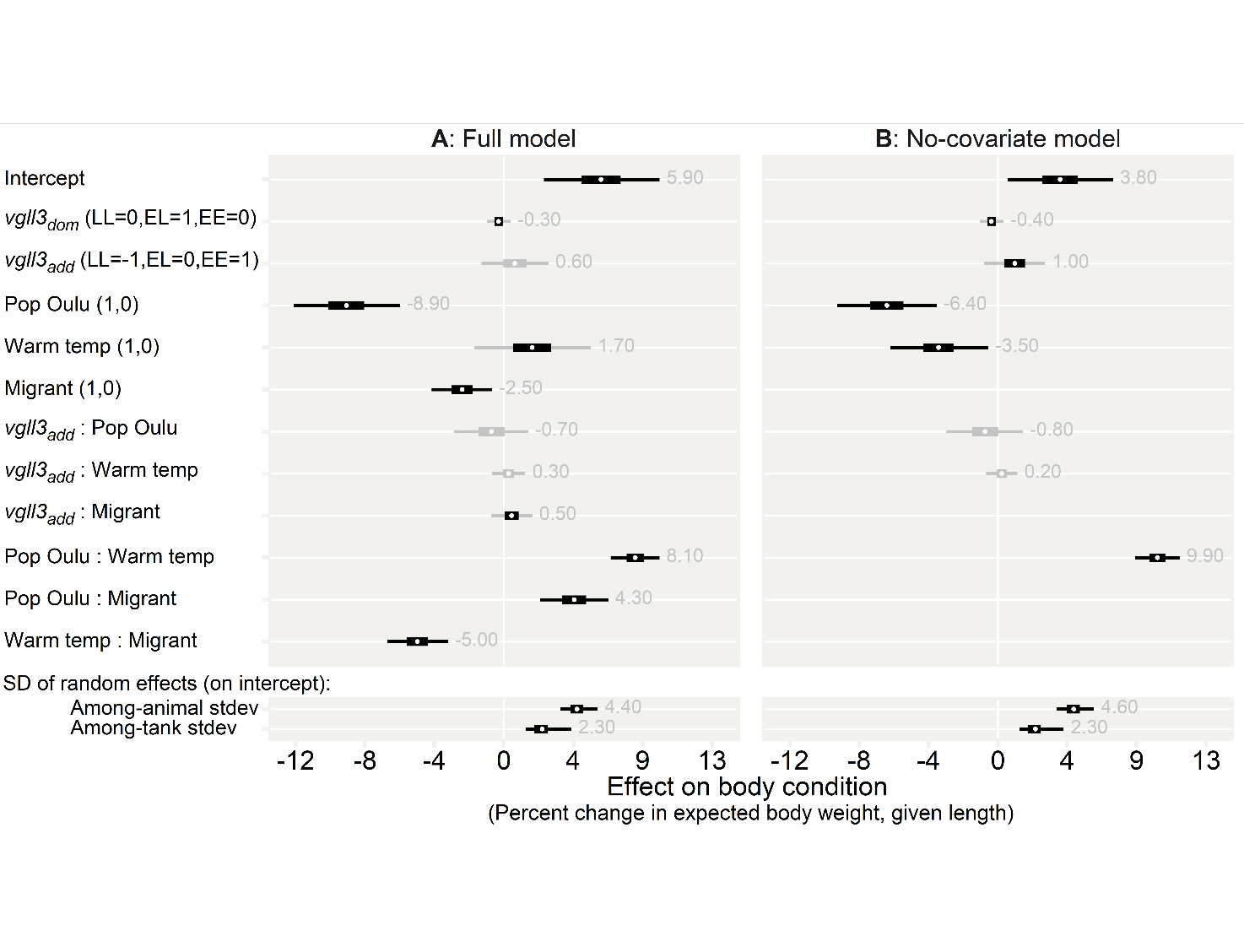


**Figure S2.2-Model-Cond.** Effect sizes (parameter estimates) for the two body condition models. A) Parameter estimates for the full model. B) A simplified model that only includes independent variables. Effects are transformed to show the percent change in expected body mass (given length), e.g., an effect size of 5 indicates that an individual is 5% heavier than what is expected for its mass. Thick and thin sections of bars indicate 97.5% and 50% credible intervals, respectively. As a visual aid, Intervals are coloured grey if they include 0. Grey numbers show the mean parameter estimate. Parentheses indicate the levels of the variables, and all variables are set to 0 for the intercept. The first level in parenthesis is the written level. The *vgll3_dom_* parameter indicates the degree of dominance displayed by either of the alleles. The lower section shows the standard deviation of the random effects, representing the degree of among-tank variation and among-animal variation (additive genetic standard deviation). The full model summaries can be found in table S2.6 and S2.7.


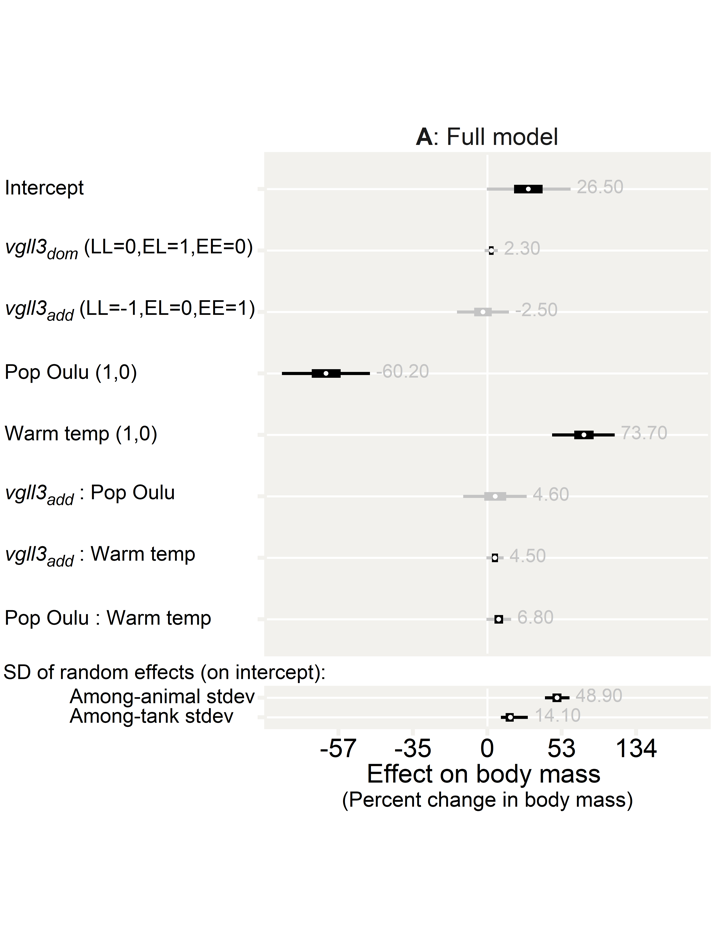


**Figure S2.3-Model-Mass.** Effect sizes (parameter estimates) for the body mass model. Effects are transformed to show the percent change in body mass. Thick and thin sections of bars indicate 97.5% and 50% credible intervals, respectively. As a visual aid, intervals are coloured grey if they include 0. Grey numbers show the mean parameter estimate. Parentheses indicate the levels of the variables, and all variables are set to 0 for the intercept. The first level in parenthesis is the written level. The *vgll3_dom_* parameter indicates the degree of dominance displayed by either of the alleles. The lower section shows the standard deviation of the random effects, representing the degree of among-tank variation and among-animal variation (additive genetic standard deviation). The full model summary can be found in table S2.8


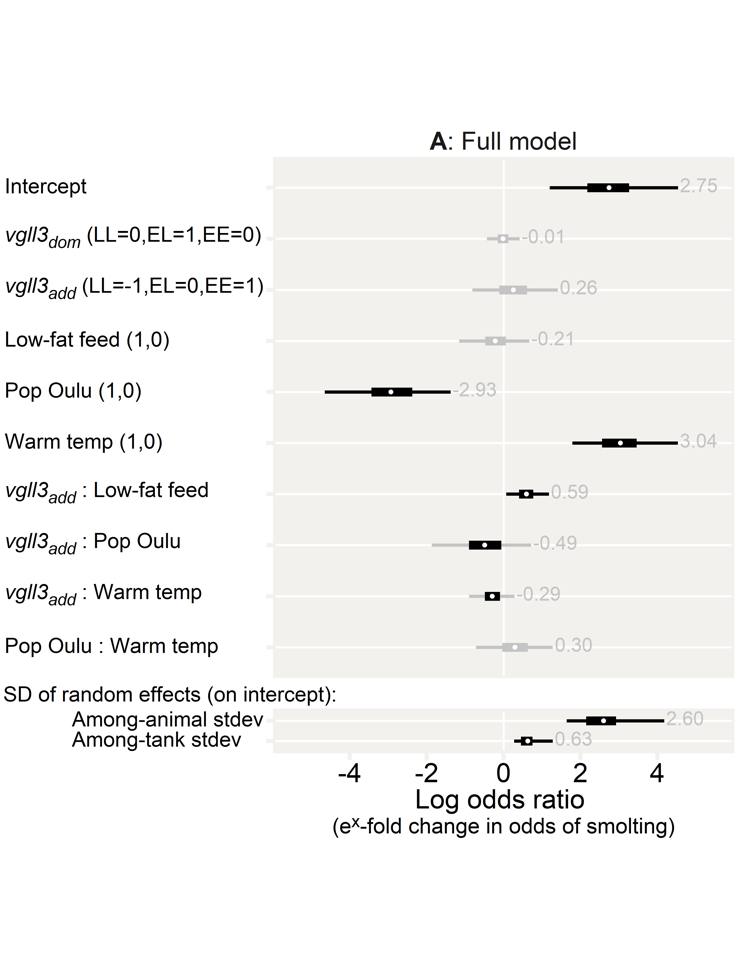


**Figure S2.4-Model-MigPheno.** Effect sizes (parameter estimates) for the migration phenotype model. Effects are shown as log odds ratios, thus indicating relative change in odds of transitioning from the resident (parr) to migrant (smolt) phenotype. Thick and thin sections of bars indicate 97.5% and 50% credible intervals, respectively. As a visual aid, intervals are coloured grey if they include 0. Grey numbers show the mean parameter estimate. Parentheses indicate the levels of the variables, and all variables are set to 0 for the intercept. The first level in parenthesis is the written level. The *vgll3_dom_* parameter indicates the degree of dominance displayed by either of the alleles. The lower section shows the standard deviation of the random effects, representing the degree of among-tank variation and among-animal variation (additive genetic standard deviation). The full model summary can be found in table S2.9.

**Table S2.4-Summary-Model-Mat-Cov.** Model summary output from *brms,* as well as pareto k diagnostic values from *loo* for the full maturation model (Model-Mat-Cov)

Family: bernoulli

Links: mu = logit

Formula: matured.5 ~ 1 + vgll3_dom + vgll3_add * (feed + population + condition.mc.sc + temp + smolted.5 + weight.log.mc.sc) + population:temp + (population + temp):(condition.mc.sc + weight.log.mc.sc + smolted.5) + (1 | gr(Animal, cov = matrix_A)) + (1 | tank)

Data: data (Number of observations: 2170)

Draws: 4 chains, each with iter = 3000; warmup = 500; thin = 1; total post-warmup draws = 10000

Group-Level Effects:

~Animal (Number of levels: 2170)

Estimate Est.Error l-95% CI u-95% CI Rhat Bulk_ESS Tail_ESS

sd(Intercept) 2.71 0.59 1.80 4.09 1.00 868 1886

~tank (Number of levels: 11)

Estimate Est.Error l-95% CI u-95% CI Rhat Bulk_ESS Tail_ESS

sd(Intercept) 0.74 0.36 0.28 1.62 1.00 2738 4809

Population-Level Effects:

Estimate Est.Error l-95% CI u-95% CI Rhat Bulk_ESS Tail_ESS

Intercept -4.27 1.08 -6.52 -2.25 1.00 5222 5469

vgll3_dom 0.41 0.29 -0.14 1.01 1.00 12133 7824

vgll3_add 2.12 0.74 0.71 3.65 1.00 8469 6272

feedLF -0.21 0.53 -1.29 0.86 1.00 8383 6705

populationOUL -0.33 1.01 -2.35 1.64 1.00 13328 7062

condition.mc.sc 0.72 0.26 0.24 1.28 1.00 5619 5196

tempwarm 5.24 0.98 3.36 7.18 1.00 3785 5935

smolted.5 -1.50 0.71 -2.94 -0.15 1.00 7067 6790

weight.log.mc.sc -0.04 0.48 -0.97 0.94 1.00 8235 7123

vgll3_add:feedLF -0.02 0.37 -0.73 0.70 1.00 12920 7687

vgll3_add:populationOUL -0.53 0.76 -2.03 0.96 1.00 10340 7452

vgll3_add:condition.mc.sc 0.44 0.17 0.12 0.79 1.00 7217 6535

vgll3_add:tempwarm 0.22 0.54 -0.81 1.29 1.00 11684 7925

vgll3_add:smolted.5 0.11 0.55 -0.96 1.23 1.00 10732 6901

vgll3_add:weight.log.mc.sc -0.14 0.35 -0.83 0.55 1.00 10678 7636

populationOUL:tempwarm -1.13 0.73 -2.59 0.28 1.00 9202 7142

populationOUL:condition.mc.sc 0.37 0.24 -0.09 0.84 1.00 7991 6675

populationOUL:weight.log.mc.sc 0.09 0.46 -0.82 1.01 1.00 8570 7719

populationOUL:smolted.5 0.03 0.72 -1.42 1.44 1.00 11535 7361

condition.mc.sc:tempwarm 0.21 0.24 -0.25 0.69 1.00 9339 6274

tempwarm:weight.log.mc.sc 2.71 0.53 1.78 3.83 1.00 2630 3500

tempwarm:smolted.5 -2.25 0.73 -3.71 -0.83 1.00 10185 6854

Draws were sampled using sampling(NUTS). For each parameter, Bulk_ESS

and Tail_ESS are effective sample size measures, and Rhat is the potential

scale reduction factor on split chains (at convergence, Rhat = 1).

Pareto k diagnostic values:

Count Pct. Min. n_eff

(-Inf, 0.5] (good) 809 37.3% 626

(0.5, 0.7] (ok) 1215 56.0% 130

(0.7, 1] (bad) 146 6.7% 31

(1, Inf) (very bad) 0 0.0% <NA>

**Table S2.5-Summary-Model-Mat-Nocov.** Model summary output from *brms,* as well as pareto k diagnostic values from *loo* for the no-covariate maturation model (Model-Mat-Nocov)

Family: bernoulli

Links: mu = logit

Formula: matured.5 ~ 1 + vgll3_dom + vgll3_add * (feed + population + temp) + population:temp + (1 | gr(Animal, cov = matrix_A)) + (1 | tank)

Data: data (Number of observations: 2170)

Draws: 4 chains, each with iter = 3000; warmup = 500; thin = 1; total post-warmup draws = 10000

Group-Level Effects:

~Animal (Number of levels: 2170)

Estimate Est.Error l-95% CI u-95% CI Rhat Bulk_ESS Tail_ESS

sd(Intercept) 2.52 0.50 1.74 3.64 1.01 808 1398

~tank (Number of levels: 11)

Estimate Est.Error l-95% CI u-95% CI Rhat Bulk_ESS Tail_ESS

sd(Intercept) 0.19 0.16 0.01 0.60 1.00 3263 4611

Population-Level Effects:

Estimate Est.Error l-95% CI u-95% CI Rhat Bulk_ESS Tail_ESS

Intercept -4.47 0.85 -6.28 -2.97 1.00 2092 2910

vgll3_dom 0.07 0.26 -0.44 0.58 1.00 7996 7714

vgll3_add 1.87 0.61 0.72 3.13 1.00 4461 4310

feedLF -0.15 0.27 -0.67 0.38 1.00 6061 4861

populationOUL -0.79 0.81 -2.40 0.76 1.00 4918 4823

tempwarm 4.88 0.64 3.79 6.29 1.00 1193 2087

vgll3_add:feedLF -0.20 0.32 -0.83 0.42 1.00 7731 6517

vgll3_add:populationOUL -0.31 0.66 -1.64 0.97 1.00 4753 5148

vgll3_add:tempwarm 0.38 0.38 -0.34 1.14 1.00 5287 6489

populationOUL:tempwarm -1.84 0.50 -2.88 -0.92 1.00 2889 3912

Draws were sampled using sampling(NUTS). For each parameter, Bulk_ESS

and Tail_ESS are effective sample size measures, and Rhat is the potential

scale reduction factor on split chains (at convergence, Rhat = 1).

Pareto k diagnostic values:

Count Pct. Min. n_eff

(-Inf, 0.5] (good) 1225 56.5% 420

(0.5, 0.7] (ok) 917 42.3% 119

(0.7, 1] (bad) 28 1.3% 415

(1, Inf) (very bad) 0 0.0% <NA>

**Table S2.6-Summary-Model-Cond-Cov.** Model summary output from *brms,* as well as pareto k diagnostic values from *loo* for the full body condition model (Model-Cond-Cov)

Family: gaussian

Links: mu = identity; sigma = identity

Formula: condition.mc.sc ~ 1 + vgll3_dom + vgll3_add * (population + temp + smolted.5) + population:temp + (population + temp):smolted.5 + (1 | gr(Animal, cov = matrix_A)) + (1 | tank)

Data: data (Number of observations: 2170)

Draws: 4 chains, each with iter = 3000; warmup = 500; thin = 1; total post-warmup draws = 10000

Group-Level Effects:

~Animal (Number of levels: 2170)

Estimate Est.Error l-95% CI u-95% CI Rhat Bulk_ESS Tail_ESS

sd(Intercept) 0.53 0.07 0.41 0.68 1.00 672 778

~tank (Number of levels: 11)

Estimate Est.Error l-95% CI u-95% CI Rhat Bulk_ESS Tail_ESS

sd(Intercept) 0.28 0.08 0.16 0.49 1.00 4119 5616

Population-Level Effects:

Estimate Est.Error l-95% CI u-95% CI Rhat Bulk_ESS Tail_ESS

Intercept 0.70 0.21 0.29 1.12 1.00 5280 5562

vgll3_dom -0.04 0.04 -0.12 0.05 1.00 12079 7932

vgll3_add 0.08 0.12 -0.16 0.32 1.00 6958 6454

populationOUL -1.13 0.20 -1.51 -0.75 1.00 8265 7013

tempwarm 0.20 0.21 -0.21 0.63 1.00 5104 5163

smolted.5 -0.30 0.11 -0.52 -0.08 1.00 10488 7496

vgll3_add:populationOUL -0.09 0.14 -0.36 0.17 1.00 6517 6948

vgll3_add:tempwarm 0.03 0.06 -0.09 0.16 1.00 12736 7871

vgll3_add:smolted.5 0.06 0.08 -0.09 0.21 1.00 11866 7541

populationOUL:tempwarm 0.95 0.09 0.77 1.12 1.00 12060 8232

populationOUL:smolted.5 0.51 0.13 0.26 0.75 1.00 10199 6748

tempwarm:smolted.5 -0.62 0.11 -0.84 -0.40 1.00 10968 7538

Family Specific Parameters:

Estimate Est.Error l-95% CI u-95% CI Rhat Bulk_ESS Tail_ESS

sigma 0.81 0.03 0.74 0.85 1.00 758 916

Draws were sampled using sampling(NUTS). For each parameter, Bulk_ESS

and Tail_ESS are effective sample size measures, and Rhat is the potential

scale reduction factor on split chains (at convergence, Rhat = 1).

Pareto k diagnostic values:

Count Pct. Min. n_eff

(-Inf, 0.5] (good) 2070 95.4% 594

(0.5, 0.7] (ok) 84 3.9% 144

(0.7, 1] (bad) 9 0.4% 26

(1, Inf) (very bad) 7 0.3% 5

**Table S2.7-Summary-Model-Cond-Nocov.** Model summary output from *brms,* as well as pareto k diagnostic values from *loo* for the no-covariate body condition model (Model-Cond-Nocov)

Family: gaussian

Links: mu = identity; sigma = identity

Formula: condition.mc.sc ~ 1 + vgll3_dom + vgll3_add * (population + temp) + population:temp + (1 | gr(Animal, cov = matrix_A)) + (1 | tank)

Data: data (Number of observations: 2170)

Draws: 4 chains, each with iter = 3000; warmup = 500; thin = 1; total post-warmup draws = 10000

Group-Level Effects:

~Animal (Number of levels: 2170)

Estimate Est.Error l-95% CI u-95% CI Rhat Bulk_ESS Tail_ESS

sd(Intercept) 0.55 0.07 0.42 0.69 1.00 629 926

~tank (Number of levels: 11)

Estimate Est.Error l-95% CI u-95% CI Rhat Bulk_ESS Tail_ESS

sd(Intercept) 0.27 0.08 0.16 0.47 1.00 2963 5053

Population-Level Effects:

Estimate Est.Error l-95% CI u-95% CI Rhat Bulk_ESS Tail_ESS

Intercept 0.45 0.19 0.07 0.83 1.00 3017 4209

vgll3_dom -0.05 0.04 -0.13 0.04 1.00 7631 7272

vgll3_add 0.12 0.11 -0.10 0.34 1.00 3519 4796

populationOUL -0.80 0.18 -1.16 -0.44 1.00 3930 4792

tempwarm -0.43 0.18 -0.78 -0.07 1.00 3086 4259

vgll3_add:populationOUL -0.09 0.14 -0.37 0.18 1.00 3310 4636

vgll3_add:tempwarm 0.03 0.06 -0.08 0.14 1.00 8980 7274

populationOUL:tempwarm 1.15 0.08 0.99 1.31 1.00 8190 7414

Family Specific Parameters:

Estimate Est.Error l-95% CI u-95% CI Rhat Bulk_ESS Tail_ESS

sigma 0.81 0.03 0.75 0.86 1.00 679 982

Draws were sampled using sampling(NUTS). For each parameter, Bulk_ESS

and Tail_ESS are effective sample size measures, and Rhat is the potential

scale reduction factor on split chains (at convergence, Rhat = 1).

Pareto k diagnostic values:

Count Pct. Min. n_eff

(-Inf, 0.5] (good) 2057 94.8% 601

(0.5, 0.7] (ok) 100 4.6% 128

(0.7, 1] (bad) 4 0.2% 45

(1, Inf) (very bad) 9 0.4% 5

**Table S2.8-Summary-Model-Mass.** Model summary output from *brms,* as well as pareto k diagnostic values from *loo* for the body mass model (Model-Mass)

Family: gaussian

Links: mu = identity; sigma = identity

Formula: weight.log.mc.sc ~ 1 + vgll3_dom + vgll3_add * (population + temp) + population:temp + (1 | gr(Animal, cov = matrix_A)) + (1 | tank)

Data: data (Number of observations: 2170)

Draws: 4 chains, each with iter = 3000; warmup = 500; thin = 1; total post-warmup draws = 10000

Group-Level Effects:

~Animal (Number of levels: 2170)

Estimate Est.Error l-95% CI u-95% CI Rhat Bulk_ESS Tail_ESS

sd(Intercept) 0.56 0.05 0.47 0.66 1.07 87 180

~tank (Number of levels: 11)

Estimate Est.Error l-95% CI u-95% CI Rhat Bulk_ESS Tail_ESS

sd(Intercept) 0.19 0.06 0.11 0.33 1.00 2821 4797

Population-Level Effects:

Estimate Est.Error l-95% CI u-95% CI Rhat Bulk_ESS Tail_ESS

Intercept 0.33 0.17 -0.00 0.67 1.00 2212 3649

vgll3_dom 0.03 0.03 -0.02 0.08 1.00 5613 7407

vgll3_add -0.04 0.11 -0.24 0.17 1.00 2628 3991

populationOUL -1.30 0.18 -1.66 -0.95 1.00 2580 3757

tempwarm 0.78 0.13 0.52 1.03 1.00 2309 3759

vgll3_add:populationOUL 0.06 0.13 -0.19 0.32 1.00 2694 4440

vgll3_add:tempwarm 0.06 0.04 -0.01 0.13 1.00 5164 6295

populationOUL:tempwarm 0.09 0.05 -0.01 0.19 1.00 4696 6715

Family Specific Parameters:

Estimate Est.Error l-95% CI u-95% CI Rhat Bulk_ESS Tail_ESS

sigma 0.36 0.04 0.27 0.43 1.08 84 161

Draws were sampled using sampling(NUTS). For each parameter, Bulk_ESS

and Tail_ESS are effective sample size measures, and Rhat is the potential

scale reduction factor on split chains (at convergence, Rhat = 1).

Pareto k diagnostic values:

Count Pct. Min. n_eff

(-Inf, 0.5] (good) 69 3.2% 242

(0.5, 0.7] (ok) 1559 71.8% 20

(0.7, 1] (bad) 523 24.1% 1

(1, Inf) (very bad) 19 0.9% 0

**Table S2.9-Summary-Model-MigPheno.** Model summary output from *brms,* as well as pareto k diagnostic values from *loo* for the no-covariate body condition model (Model-MigPheno)

Family: bernoulli

Links: mu = logit

Formula: smolted.5 ~ 1 + vgll3_dom + vgll3_add * (feed + population + temp) + population:temp + (1 | gr(Animal, cov = matrix_A)) + (1 | tank)

Data: data (Number of observations: 2170)

Draws: 4 chains, each with iter = 3000; warmup = 500; thin = 1; total post-warmup draws = 10000

Group-Level Effects:

~Animal (Number of levels: 2170)

Estimate Est.Error l-95% CI u-95% CI Rhat Bulk_ESS Tail_ESS

sd(Intercept) 2.60 0.64 1.65 4.18 1.01 545 974

~tank (Number of levels: 11)

Estimate Est.Error l-95% CI u-95% CI Rhat Bulk_ESS Tail_ESS

sd(Intercept) 0.63 0.26 0.28 1.28 1.00 2383 3600

Population-Level Effects:

Estimate Est.Error l-95% CI u-95% CI Rhat Bulk_ESS Tail_ESS

Intercept 2.75 0.84 1.20 4.54 1.00 4484 2929

vgll3_dom -0.01 0.21 -0.43 0.42 1.00 9660 6069

vgll3_add 0.26 0.55 -0.81 1.41 1.00 6499 5490

feedLF -0.21 0.45 -1.16 0.67 1.00 6249 4893

populationOUL -2.93 0.83 -4.65 -1.37 1.00 4674 4547

tempwarm 3.04 0.70 1.79 4.53 1.00 1572 2118

vgll3_add:feedLF 0.59 0.28 0.07 1.18 1.00 5028 4332

vgll3_add:populationOUL -0.49 0.65 -1.87 0.72 1.00 5447 3282

vgll3_add:tempwarm -0.29 0.30 -0.89 0.28 1.00 7528 5635

populationOUL:tempwarm 0.30 0.51 -0.72 1.28 1.00 7783 6637

Draws were sampled using sampling(NUTS). For each parameter, Bulk_ESS

and Tail_ESS are effective sample size measures, and Rhat is the potential

scale reduction factor on split chains (at convergence, Rhat = 1).

Pareto k diagnostic values:

Count Pct. Min. n_eff

(-Inf, 0.5] (good) 1134 52.3% 378

(0.5, 0.7] (ok) 986 45.4% 64

(0.7, 1] (bad) 50 2.3% 61

(1, Inf) (very bad) 0 0.0% <NA>

**Table S2.10.** Observed maturation of different combinations of maturation- and migration phenotypes (migrant=smolt, resident=parr) for male *Salmo salar*  in the winter of their third year (early 2020) post-fertilization, for combinations of temperature treatment and population of origin.

| Group | n | Matured-migrant | Matured-Resident | Immature-Migrant | Immature-Resident |
| --- | --- | --- | --- | --- | --- |
| Neva, Cold | 405 | 6.9% | 2.7% | 76.8% | 13.6% |
| Neva, Warm | 430 | 52.3% | 1.9% | 44.7% | 1.2% |
| Oulu, Cold | 749 | 1.9% | 3.2% | 42.6% | 52.3% |
| Oulu, Warm | 586 | 17.7% | 6.0% | 67.1% | 9.2% |
| Neva | 835 | 30.3% | 2.3% | 60.2% | 7.2% |
| Oulu | 1335 | 8.8% | 4.4% | 53.3% | 33.4% |
| Cold | 1154 | 3.6% | 3.0% | 54.6% | 38.7% |
| Warm | 1016 | 32.4% | 4.2% | 57.6% | 5.8% |
| Total | 2170 | 17.1% | 3.6% | 56.0% | 23.3% |

SUPPLEMENTARY MATERIAL 3: Supplementary data


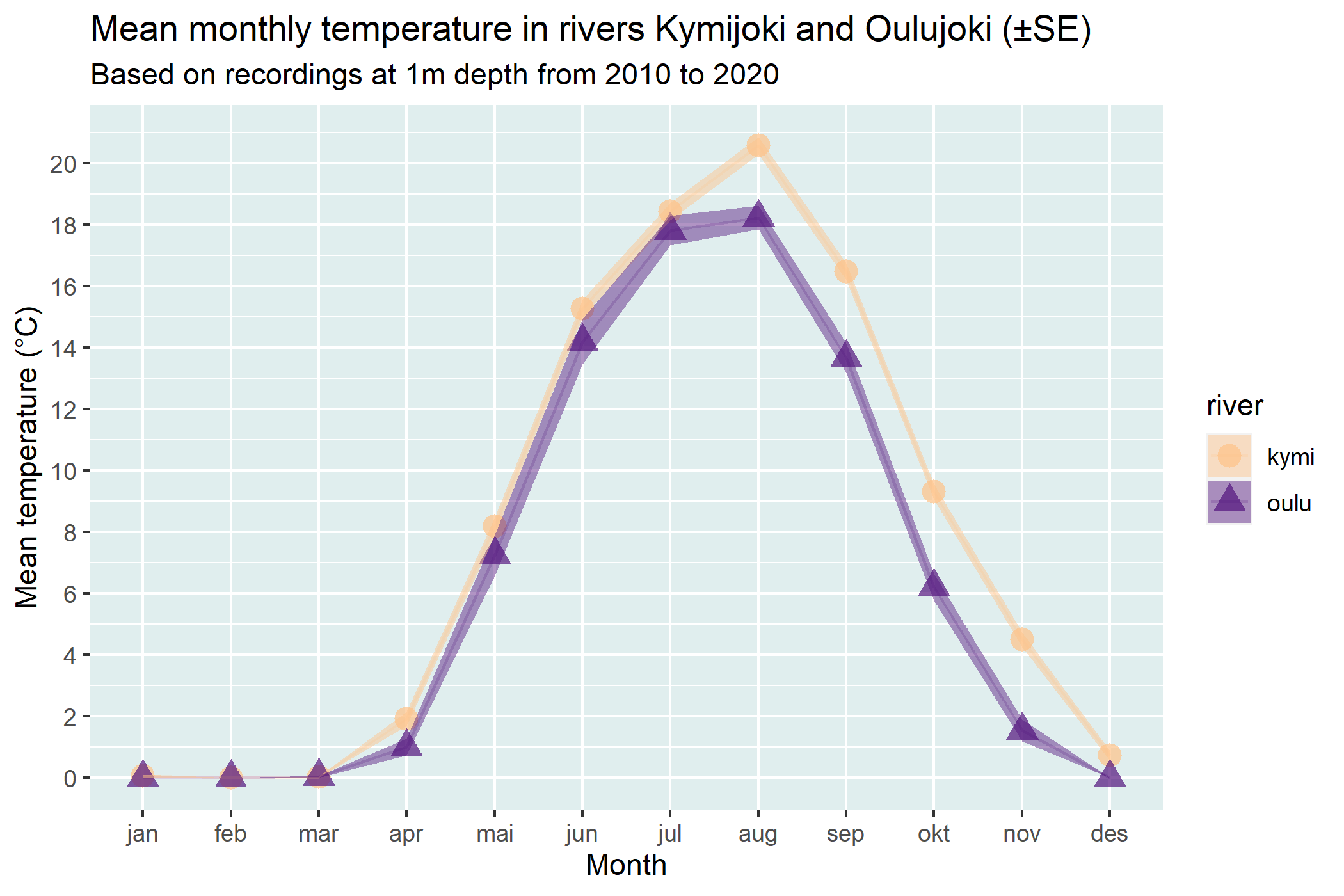


**Figure S3.1-River-temperatures.** Shows average temperatures for each month over multiple years for multiple locations in rivers Kymijoki (orange, circles) and Oulujoki (purple, triangles). All temperature recordings are made at 1 m depth. Sites are shown in Figure S3.2-Map. Temperature data aquired from the Finnish Environment Institute’s (SYKE) Hertta system.


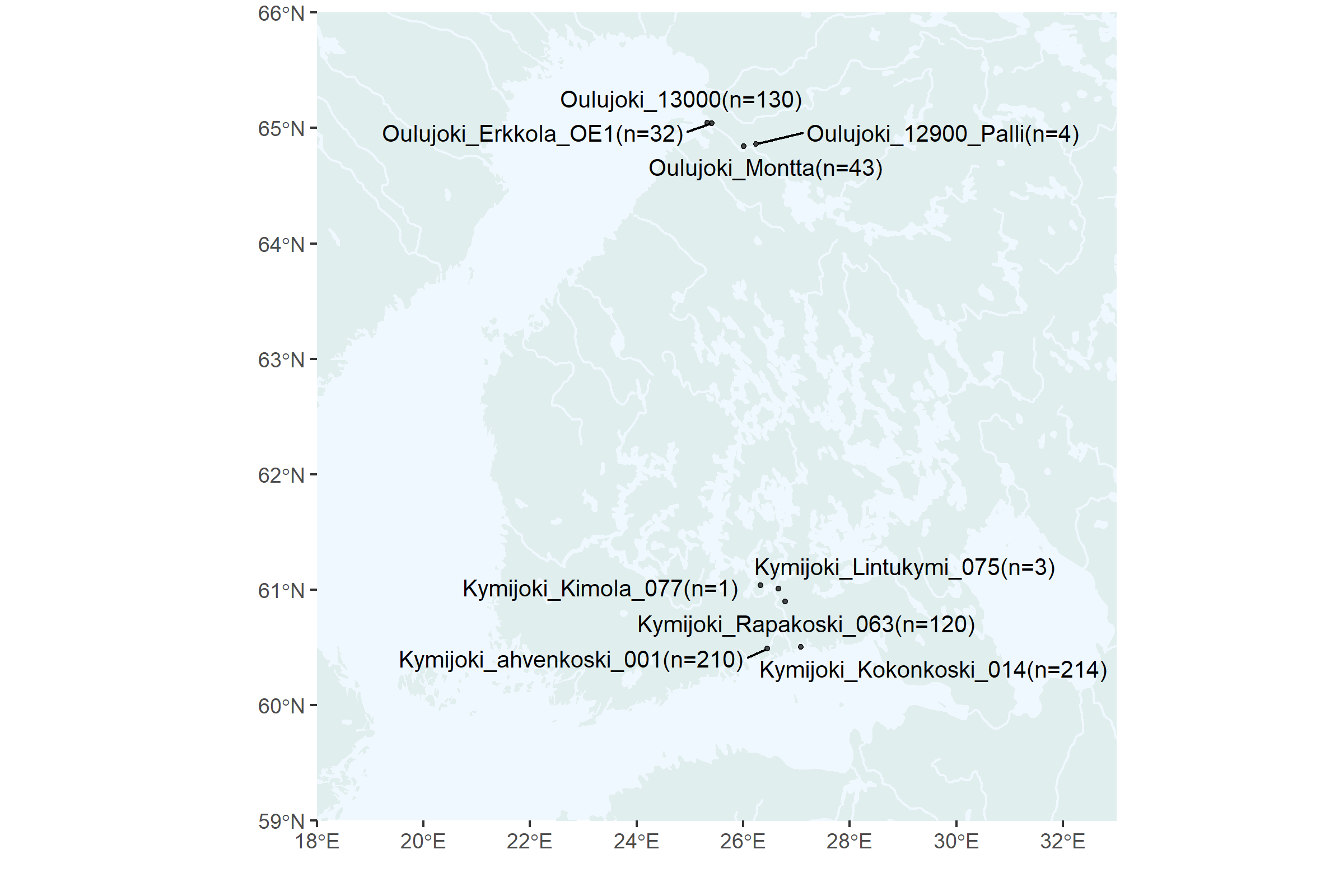


**Figure S3.2-Map.** Shows sites of temperature recordings for Figure S3.1-River-temperatures. Numbers in parantheses indicate number of temperature observations from each site.
